# An exploration of the conditions for deploying self-management strategies: a qualitative study of experiential knowledge in depression

**DOI:** 10.1186/s12888-020-02559-3

**Published:** 2020-05-11

**Authors:** Dorien Smit, Janneke Peelen, Janna N. Vrijsen, Jan Spijker

**Affiliations:** 1 Behavioural Science Institute, Radboud University Nijmegen, PO Box 9101, Nijmegen, 6500 HB The Netherlands; 2 Pro Persona Mental Health Care, Depression Expertise Center, PO Box 7049, Nijmegen, 6503 GM The Netherlands; 3 HAN University of Applied Sciences, Research Group for Key Factors in Youth Care, PO Box 6960, Nijmegen, 6503 GL The Netherlands; 4 Department of Psychiatry, Donders Institute for Brain, Cognition and Behaviour, Radboud University Medical Center, PO Box 9010, Nijmegen, 6500 GL The Netherlands

**Keywords:** Depression, Self-management, Experiential knowledge, Empowerment, Qualitative research, Patients’ perspectives

## Abstract

**Background:**

Living with recurrent, and/or chronic depression requires long-term management in addition to active coping on a day-to-day basis. Previous research on long-term management, and coping with depression mainly focused on identifying self-management strategies. However, research on the conditions for deploying self-management strategies in depression is lacking. By means of exploring the development of experiential knowledge in depression, and its relation with coping with depression, this study aims to gain insight into the conditions for deploying self-management strategies.

**Methods:**

In the current qualitative study, individual pathways to recovery, living with depression, and recurrence risk were assessed, including but not limited to long-term management. ‘Experiential knowledge’, which can be defined as patients’ unique knowledge and own lived experiences in facilitating and debilitating factors in the recovery process and coping with the disorder, was used as a sensitizing concept. Thirteen semi-structured interviews were conducted with individuals who experienced at least two depressive episodes and were currently in (partial) remission, plus two deviant cases were interviewed to check for saturation. Until saturation was achieved, participants were purposively selected to include diverse perspectives on coping with depression. Data were analysed using a narrative research method.

**Results:**

The results show that deploying self-management strategies are an integral part of ‘experiential knowledge’. The evolvement of experiential knowledge can be seen as a cyclical process of the main themes that were identified as relevant when coping with depression: introspection, empowerment, self-management strategies, and external moderators of the environment. The identification of supporting and impeding factors in coping with depression from a patient perspective might increase a sustainable use of self-management strategies.

**Conclusion:**

These results highlight the need for an individualised holistic model of coping with depression, both in research, and in practice. By means of integrating experiential knowledge in this holistic approach, the conditions for deployment of self-management strategies in depressive patients can be specified.

## Background

Depression is a growing global health problem, associated with significant social and physical disability, mortality, and economic burden [30]. It is considered to be a chronic illness: recurrence rates are high around 60% after 5 years, rising to 85% after 15 years [17, 31]. Almost 20% of all patients develop chronic depression (≥ 2 years of symptoms) [16, 33]. Moreover, approximately 50% of depressed patients respond insufficiently to treatment [15, 28]. The chronic nature as well as suboptimal treatment response is indicative of the fact that depression requires long-term management [13, 23, 34].

In recent years the view on mental disorders has shifted from a primary focus on symptom reduction to including the acknowledgement of the importance of coping with problems related to the mental disorder [19, 22, 35]. Previous studies on coping with depression concentrated on the concept of self-management [2]. Self-management is defined as ‘the training, skill acquisition, and interventions through which patients who suffer from a disease or chronic condition may take care of themselves and manage their illnesses’ [38]. Specifically, research has been aimed at identifying self-management strategies. Engaging in sports activities and having a good day and night rhythm are examples of strategies that are perceived as helpful when coping with depression [26, 36, 37]. Collectively, the results indicate that deploying self-management strategies can provide a positive contribution to recovery and coping with depression, such as lower depressive symptoms, an improvement of self-efficacy, and empowerment [20, 22, 27]. However, the way in which self-management strategies in depression treatment are dealt with has been criticised as being overly simplistic, because symptoms of depression (such as passive behaviour) can interfere with self-management [14, 18]. Moreover reduced self-confidence and energy, and/or an increased state of confusion caused by the many choices in strategies can impede the use of self-management [14, 29]. In fact, the inability to engage in self-management can lead to feelings of helplessness and hopelessness [13, 28], which contributes to the depression [29]. Therefore, more knowledge about the implementation of coping with recurrent and/or chronic depression, including the conditions for deploying self-management strategies, is of major importance.

The use of *experiential knowledge* is an important feature in coping with a somatic or mental disorder. The concept of experiential knowledge refers to patients’ unique knowledge and own lived experiences in helping and debilitating factors in the recovery process and coping with the disorder [3, 6, 9, 37]. There is a relatively small body of literature concerning experiential knowledge. To date, studies focused on the effect of deploying experiential knowledge by adding peer support to treatment as usual for individuals with chronic illnesses, such as somatic disorders and physical disability [9, 39, 40], as well as psychotic disorders and trauma exposure [5, 34, 41]. Although some positive effects on hope, recovery, and empowerment were found, these results should be interpreted with caution because of the limited number of studies, the complexity, and variety of interventions [41]. Moreover, there is a lack of clarity about the substantive themes that are associated with the concept of experiential knowledge. Hence, a deeper understanding of experiential knowledge, in which helping as well as debilitating factors in coping with the disorder are defined, could shed light on the conditions for deploying self-management strategies. Research on experiential knowledge in depression is currently lacking. To give an in-depth description of coping with recurrent and/or chronic depression, the concept of experiential knowledge should be examined.

This qualitative study aims to explore the relation between the development of experiential knowledge and coping effectively with depression, as well as the conditions for deployment of self-management strategies. Semi-structured interviews were conducted in a heterogenic group of individuals who have experience with depression (> 2 past episodes).

## Methods

### Design and participants

A qualitative semi-structured interview study design was used. Experiential knowledge was approached as a sensitizing concept. As explained by Boeije [4] sensitizing concepts start out with a general description, because they are not yet specified and clarified in the research field. In this research, the general description of experiential knowledge was used as a guiding framework for exploring an in-depth characterization of the construct from a patients’ perspective. Fifteen face-to-face interviews, with open-ended questions about personal experiences in coping with the illness, were conducted with individuals who experienced at least two depressive episodes over the course of 3 years minimum, and were currently in (partial) remission. Members of the Dutch Depression Association were approached for participation. Furthermore, recruitment took place via national and regional news websites about mental health care. Data collection continued until saturation was reached, that is when no new topics emerged during the interviews. To check for saturation, deviant cases were included; one individual with a current depression, and one individual who experienced a single depressive episode.

Participants had to meet the following criteria: 1) two or more past depressive episodes (except for one deviant case), 2) the first depressive episode had occurred at least 3 years prior to participation. These criteria were used to ensure participants’ ability to reflect on their depressive experiences during the interviews. The following exclusion criteria were used: age younger than 18 years, a current depressive episode (except for one deviant case), bipolar-, or (a history of) psychotic disorder, current drug abuse, and current severe risk of suicidality. Eligibility was assessed by telephonic administration of the brief structured diagnostic interview MINI [Mini International Psychiatric Interview for DSM-IV-TR] [25, 32].

Throughout the entire duration of this study, thirty-four people were interested in participating in the interview study. Participants with diverse clinical and demographic characteristics (e.g. number of depressive episodes, age, ethnicity, educational level) were included to increase conceptual variation. These characteristics were examined by telephonic screening before conducting the MINI interview. As a result, sixteen people were not included. Moreover, according to the exclusion criteria three participants were excluded due to a current depression, drug abuse or the experience of several psychoses during depressive episodes. This resulted in fifteen study participants (eight men, seven women; including two deviant cases). Table 1 shows the participants’ demographic and clinical characteristics.

**Table 1:** Demographic and clinical characteristics of participants (*n* = 15)

Gender, *n*	
Male	7
Female	8
Membership Dutch Depression Association	
Yes	7
No	8
Age, *years*	
Mean (SD)	43,5 (15)
Range	23–67
Ethnicity, *n*	
Dutch	11
Surinamese/Antillean	1
Serbian/Croatian	1
Surinamese/Hindustani	1
Dutch Antillean	1
Educational level, *n*	
Secondary education	3
Secondary Vocational Education and Training	1
Higher education (research-oriented and profession-oriented)	11
Treatment history type^a^, *n*	
A form of therapy in mental health care (i.e. CBT, psychodynamic psychotherapy)	15
Former use of medication	5
Current use of medication	7
Never used medication	3
Number of depressive episodes^a^, *n*	
One episode (negative case)	1
Two episodes	1
3–5 episodes	7
> 5 episodes	4
Chronic course only (≥ 2 years of symptoms)	2
*Chronic course in addition to depressive episodes*	*4*
Age at onset, *years*	
Range	12–45
12–18	5
19–25	3
26–32	3
33–45	3
≥ 46	0
Unknown	1
Years since onset, *years*	
Range	10–45
0–10	2
11–20	4
21–30	6
31–40	2
41–50	1

^a^Including overlap

The local ethics committee [Commissie Mensgebonden Onderzoek Arnhem-Nijmegen] assessed the research protocol for this study. According to the regulations of the Medical Research (Human Subjects) Act, they stated that further approval was not deemed necessary given the minor burden of participation in this study.

### Interviews

Confidentiality was guaranteed to the participants. Information about the study was given in oral and written form. Informed consent was signed before starting the interview. In the semi-structured interview, participants’ underlying ideas of behaviour, choices and thoughts in coping with depression were explored. The main question in the interview was “*What did you learn while living with depression?”*. To ensure that the main topics of the research question were discussed by all participants, an interview guide was produced based on literature, preliminary consultation and orienting interviews with social scientist researchers, a psychiatrist, and patients. The topics were clustered in five discussion topics, which are presented in Table 2. The interviews were conducted by one researcher (author DS). The interview guide was modified after finishing the third, fifth, ninth and eleventh interview, so that new emerging topics could be further explored.

**Table 2:** Topics interview guide in keywords^a^

Main discussion topics	Subtopics
Course of the disorder	Experiences depressive episodes, triggers, development of the depression
Coping with depression	Dealing with the illness, practical skills, personal characteristics, supportive network
Self	Self-reflection, influence of experiencing depression on identity
Experiential expertise	Opinion on the role of experiential knowledge and -expertise in mental health care
Mental health care	Experiences with treatment for depression

^a^Complete interview guide available

Interviews were conducted between May 2018 and August 2018. The average duration of an interview was 73,6 min (Range: 45–89 min; SD: 13,9 min). When participants were perceived to be in psychological stress or reported discomfort, ending or pausing the interview was suggested. Although no interview was ended prematurely because of participants’ distress, one interview was temporarily paused because of participants’ emotional experiences. All interviews were audio recorded and transcribed verbatim into written text as accurately as possible, including pauses, and non-verbal sounds. Identifying information such as the names of individuals were removed during the transcription process.

### Analyses

Data were qualitatively analysed in accordance with a narrative research method, focusing on the perspective of the information (who said it), what and how it is narrated [1]. Atlas.ti software (version 8) was used for the coding process. After the first two interviews two researchers (authors DS and JP) examined the transcripts. This open coding process began with line-by-line microanalysis aimed at identifying categories within the data. The two researchers (DS, JP) used independent coding to ensure inter-coder reliability. The researcher (DS) continued the analysis progressing to axial coding, by condensing codes, exploring categories, their properties, and the relationships between them [4]. To ensure the validity of the ongoing data analysis, the process of coding was discussed with two independent researchers. Interim findings informed the process of purposive sampling, and adjustments of the interview guide.

In addition, two member checks, after the analysis of eight and subsequently eleven interviews, were held to validate the analysis. These meetings were attended by respectively three and four participants. Also, to discuss study results from a broad perspective, a focus group was conducted after finalisation of data collection. This meeting was held with six independent experiential experts (non-participants, recruited via diverse websites for mental health care and the Dutch Depression Association) and four health care professionals of depression (psychologists and psychiatrists, recruited via Radboud University Medical Centre and Pro Persona Mental Health Care). By initiating a group discussion to reflect on the results of the qualitative study, the interim findings were validated, no new main themes were found. However, a lack of attention for physical wellbeing in mental health care, and limited focus on involving relatives in treatment were brought up as underexposed factors.

Concerning the influence of the researcher to the interview process and to the participants, a reflexive logbook was kept. Limited clinical and personal experiences with depressive patients and the absence of any formal role (regarding treatment) between participant and researcher, aided to remain open and contributed to an objective stance.

## Results

### The development of experiential knowledge

Results indicate that experiential knowledge evolves from three intrapersonal levels: 1) In a process of *introspection*, 2) in the development of *empowerment*, and 3) in learning and deploying *self-management strategies*. Finally, external moderators of the evolvement of experiential knowledge seem to appear at an interpersonal level, which are described under 4) *the environment*. Constant interaction between these three intrapersonal levels as well as interaction between an interpersonal process and intrapersonal factors is observed in the data. The main themes as well as subthemes derived from the narratives are presented in Table 3. Excerpts from various respondents are used throughout the presentation of results. The quotes are illustrative of the complex, ongoing development and interaction of different aspects in coping with depression. Together they should provide the reader with a comprehensive picture of the developmental pathway of experiential knowledge. Participants are numbered randomly (P1, P2, etc.).

**Table 3:** The development of experiential knowledge in long-term depression

Main themes of experiential knowledge	Subthemes
1. Introspection	Self-reflection
	Self-compassion
	(Self-)acceptance
	Meaning-making
2. Empowerment	Autonomy
	Self-confidence
	Future perspective
3. Self-management strategies	Daily schedule/structure
	Activities
	Self-help
	Contact with others
4. The environment	Societal context
	Mental health care
	Social support

#### Introspection

The evolvement of experiential knowledge seems to start with the realization of ‘disconnection’. Data revealed that nearly all participants lost connection with themselves, with others, and with society, while suffering from depression. Feelings of loneliness were reported. Three depressive patients described this disconnection as *‘wearing a mask’*, explained as misrepresenting oneself by hiding or neglecting negative feelings:

Woman, 33 years old, Dutch Antillean: *“Actually, I was wearing a mask, and I kept pushing forward. That is what exhausted me at the end of the day, because I had to pretend I was okay, while sometimes I did not manage to carry on.”* (P6).

When participants managed to get rid of their ‘masks’ they mentioned a responsiveness to learn about their own character, background, desires, strengths, and vulnerabilities. This process is referred to as introspection. Breaking down the concept of introspection, four processes can be discerned: self-reflection, self-compassion, (self-)acceptance, and meaning-giving.

*Self-reflection* entails the personal examination of the own conscious thoughts and feelings, which increase the understanding of relapse triggers:

Man, 27 years old, Dutch Antillean: *“By discovering my main three negative thoughts, I was able to recognize that the other thousands negative thoughts were linked to them. A very organised list of thoughts was formed, which cleared my mind because I had the common sense to understand what my triggers were.”* (P8).

More than half of the participants mentioned *self-compassion* as important in coping with depression. Being kind, attentive and patient with oneself, and to ignore the critical inner voice or expectations of others, are mentioned as facilitators in dealing with the disorder:

Woman, 25 years old, Dutch: *“If you can accept that normal activities in life are difficult to succeed when you are depressed, you can let go of the pressure and expectations towards yourself. So, you won’t disappoint yourself all the time.”* (P11).

*(Self-)acceptance* comprises the acceptance of depression as an illness and acceptance of the self, with one’s positive and negative characteristics, as is intertwined with self-compassion:

Man, 46 years old, Dutch: *“For me, acceptance is feasible by means of meditation. Because of the mild attitude and gentleness: “anything goes”. Before, I was fighting against all those negative thoughts, the opposite of accepting emotions. I attended a mindfulness and compassion-training. The gentleness is healing and lowers the impact of negative thoughts and emotions.”* (P2).

To develop experiential knowledge by means of acceptance, half of the participants suggested that it is crucial to consider depression as a disease, and explicitly not to consider depression to be an integral part of one’s identity:

Man, 23 years old, Dutch: *“I consider it as an illness which can be dealt with. When you consider depression as a part of your identity, it will be very difficult to manage because actually, you are fighting against yourself. That doesn’t make sense.”* (P1).

Finally, *meaning-making* entails the active engagement in the act of making sense of living with depression. The meaning that participants attributed to depression was unanimously described as increased self-knowledge, a desire to help fellow sufferers, and a grateful attitude in life:

Woman, 38 years old, Serbian/Croatian: *“Many times, I have said coping with depression enriched me. I do not want to experience it all over again. However, I discovered things that I did not notice before, or was not able to appreciate. In fact, I live a more conscious and a - somewhat overstated - grateful life.”* (P3).

Whereas acceptance of depression appears to be a necessity for meaning-making, it is considered very difficult by four participants, mainly because of the caused misery in their life:

Man, 27 years old, Dutch Antillean: *“Perhaps, I find acceptance overstated, because I hate it when I feel it is kicking in. When you lived with depression for nine years, you know how it feels, it can torment you to the bones. I can recognize the depression, but really accept it, no.”* (P8).

Taken together, a deepened self-understanding by means of introspection appears to be of major importance to develop experiential knowledge. In fact, the more an individual knows about the self, adopts a mild attitude, and develops acceptance towards the depression, the more personal experiential knowledge arises on how to effectively deal with the illness.

#### Empowerment

The data revealed three competencies associated with empowerment: autonomy, self-confidence, and having a future perspective. In the interviews, all participants described regaining individual responsibility and grip on life, i.e. *autonomy* as a tipping point in the capability to manage the depression in a healthy way:

Woman, 38 years old, Serbian/Croatian: *“I used to give responsibility to someone else ( …*). *I blamed my family, my friends, my illness. I always thought things happened to me, that I was not in control. Then, I realised you can create your own life, there is always a choice. You do not have to wait for support or… you just have to do it yourself. At that point, I knew I was strong enough to take care of myself and make my own decisions.”* (P3).

A third of the participants explained that being autonomous requires courage; (new and/or individual) choices are often about taking risks. This explanation shows that autonomy is interrelated with *self-confidence*. Both competencies are considered to be important as it helps to pursue personal values and wishes, instead of adjusting to the barriers of depression or wishes of others. The data show that it takes a considerable amount of positive events in daily life to develop autonomy and self-confidence, i.e. a feeling of trust in one’s abilities, qualities, and judgement:

Man, 67 years old, Dutch: *“Try something new, something very small and insignificant. For example, change your sandwich filling. If you do enough of these new, little things, a feeling of fulfillment and a bigger shift of perspective may be the result. Hence, you could break the vicious circle of the depression ( …*). *Moreover, you feel more autonomous, making your own choices gives you a feeling of control, you will feel more alive.”* (P7).

Furthermore, thinking and acting based on a *future perspective* facilitates empowerment. It contributes to personal fulfilment. Having a purpose in daily life, such as taking care of children or relatives, having an interesting job or looking after pets are important in developing this competency:

Woman, 44 years old, Dutch: *“For me, work is the best way for recovery. It gives meaning in life, and a sense of fulfilment. Yes, work keeps me going.”* (P12).

Empowerment involves the ability of an individual to make one’s own choices, which are in line with personal needs, a belief in oneself, and a future perspective. Feeling empowered seems to be of practical significance in the evolvement of experiential knowledge, because results suggest that this competency facilitates coping adequately with depression.

#### Self-management strategies

Experiential knowledge, specified as empowerment and introspection, appears to unfold by means of self-management strategies. Respondents referred to self-management strategies as practical coping skills when managing the challenges of depression on a day-to-day basis, including the risk of a relapse. The narratives indicate that living with recurrent and/or chronic depression requires a long-term deployment of self-management strategies in life. Whereas dealing with the symptoms of a current depression involves a more acute implementation of self-management strategies that are meant to control the condition:

Woman, 60 years old, Dutch: *“In the “red phase”, when a depressive episode starts, I take as much rest as possible and some extra medicine. I need sufficient rest, enough sleep, and no stress. A daily structure helps because it is important to respect my own boundaries, physically and mentally (…*). *Next, in the “orange phase”, it is important to be aware of my emotions and to meet someone who listens to me. Talking helps to organise my thoughts. At the last stage, “green”, it is important to think about the future, to do pleasurable activities and to engage in social contact with others. For me these things are impossible in the first stages.”* (P9).

Strategies might be divided into acute self-management strategies: daily schedule or structure and engaging in activities, in addition to long-term self-management strategies: self-help and contact with others. First, creating a *daily schedule or structure* was indicated as an effective self-management strategy at the initial stage of a depressive episode. It helped respondents to take rest and acknowledge depressive symptoms. In addition to planning their daily lives, participants suggested taking medication, sleep, and staying in familiar surroundings. Secondly, engaging in *activities* was described as doing pleasant and low-threshold activities in a familiar environment. Activities that involved social interaction and exercising were especially helpful, as these activities contributed to self-confidence. The long-term self-management strategy *self-help* helped participants to engage in self-reflection and taking rest. Interviewees mentioned self-help methods such as writing about personal experiences, meditation, mindfulness, reading inspiring self-help books or watching YouTube movies about personal development. Finally, participants indicated the strategy to establish or maintain *contact with others,* contributing to a sense of belonging. Respondents emphasised the importance of openness and an equal nature of social interaction. As follows from the data, the deployment of self-management strategies interacts with the evolvement of empowerment and introspection, i.e. experiential knowledge. It seems that both categories of self-management strategies need to be adjusted to the personal context of depression in order to be meaningful. As follows from the data, the deployment of self-management strategies interacts with the evolvement of empowerment and introspection, i.e. experiential knowledge:

Man, 67 years old, Dutch: *“If you want to offer resistance against depression, you need three stages in my experience. To begin with, you have to be kind to yourself. Try to find activities that give a little bit of pleasure. The second stage consists of a mindful attitude towards your environment, a gentle lens. So, if you are outside, maintain a moment-by-moment awareness of your surrounding environment, for example by looking at the clouds. Then, stage three is the opposite of this gentle attitude. When you are recovering, you have to demand yourself to do things because it will give a good feeling to succeed.”* (P7).

In summary, carrying out self-management strategies seems to be a manifestation of experiential knowledge, and thus introspection and empowerment. In this process, more knowledge about dealing effectively with the illness has arisen.

#### Important external moderators: the environment

In this research, the focus on the evolvement of experiential knowledge lies on intrapersonal levels. However, the data also reveal that the evolvement of experiential knowledge cannot be fully understood without acknowledging the complex set of influential factors in the environment of the individual which unfold at an interpersonal level. Factors in the environment are clustered in the societal context, mental health care, and social support systems. They individually and interactively influence the processes at an intrapersonal level, i.e. introspection, empowerment, and self-management strategies.

When discussing the *societal context,* the experience of discrimination and stigma was mentioned as an impeding factor of coping with depression by *t*hree-quarters of the respondents. While openness about depressive feelings is perceived to be helpful in coping with the disorder:

Man, 23 years old, Dutch: *“We never spoke about it at home. My parents knew there was something wrong, but they could not see what it was. For me, that was very difficult because of the loneliness. In retrospect, discussing it openly was good. In my experience, depression is still a taboo subject. As, if I said “I have asthma”, there is no taboo at all.”* (P1).

In *mental health care*, particularly developing self-reflection is mentioned as contributing to the development of experiential knowledge:

Man, 62 years old, Dutch: *“Self-reflection is not ‘just there’. It took many years to develop. With the help of individual treatment in mental health care, my self-knowledge increased by leaps and bounds.”* (P15).

Thirdly, the importance of *social support* from peers, family and friends in the evolvement of experiential knowledge is reflected in all narratives:

Woman, 55 years old, Dutch: *“There were friends who saw me as an independent person, ignoring the impact of the depression on my appearance. My friends made positive change. They kept supporting me, they kept believing in me, treated me positively. The connection I felt with this group of friends lead to a feeling of acceptance, which had a positive impact on my day-to-day functioning.”* (P10).

The data reveals that the environment can stimulate or impede coping with depression as well as the evolvement of experiential knowledge. The ability to deal effectively with the disorder can be influenced by various factors in the environment, which are unique per individual.

## Discussion

### Main findings

**Fig. 1:**
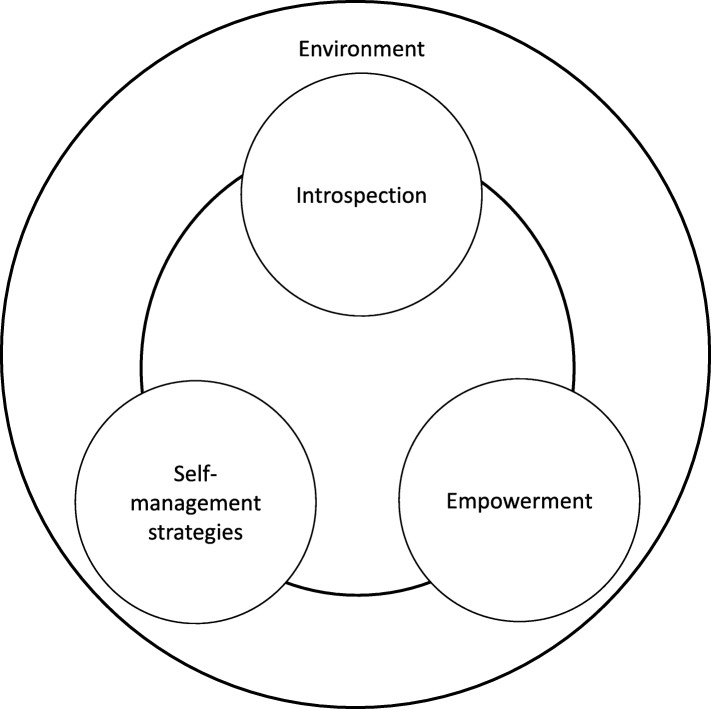
The development of experiential knowledge in recurrent and/or chronic depression

The current study examined the evolvement of experiential knowledge in depression. The relation between experiential knowledge and coping effectively with depression, as well as the conditions for deployment of self-management strategies when coping with depression were explored. The results show that from the patients’ perspective, experiential knowledge evolves from three intrapersonal levels: introspection, empowerment and self-management strategies, and one interpersonal level: the environment. Specifically, the data show a continuous interaction among the intrapersonal levels and interpersonal level of experiential knowledge, suggesting that the evolvement is a cyclical process, as shown in Fig. 1.

Experiential knowledge, in terms of introspection, empowerment, self-management strategies, and facilitating and impeding factors in the environment, seems to contribute to how well patients can cope with depression. As follows from the data, it could be hypothesised that a positive interaction between these processes lead to the sustainable deployment of self-management strategies. Firstly, through a deepened self-understanding by means of introspection, patients might more easily determine self-management strategies that match their current condition, overall character, triggers for depression, and coping style. Secondly, when empowerment develops, the patient’s ability to make one’s own choices in coping with depression may increase self-management strategies that fit the patient. When strategies are in accordance with personal preferences, this might lead to a long-term deployment. Thirdly, there seems to be a bidirectional relation between the evolvement of experiential knowledge and deployment of self-management strategies. This implies both that self-management strategies are central to the development of experiential knowledge, and that a sustainable deployment of self-management strategies in turn requires increased introspection and empowerment. Finally, it could be assumed that a sustainable deployment of self-management strategies may be enhanced by adjusting self-management strategies to facilitating and impeding factors in patient’s unique environment.

### Findings in context

In accordance with previous research on experiential knowledge in psychotic disorders and trauma exposure [5, 9, 13, 38], the results of this study highlight that experiential knowledge results from a combination of cognitive and emotional experiences, and patients’ practical coping skills in daily life [7, 10, 12]. The synergetic relationship between the processes in the evolvement of experiential knowledge implies that introspection, empowerment, and factors in the environment must be addressed when developing a personal array of self-management strategies. Stimulating the evolvement of experiential knowledge of an individual patient may be helpful to exceed personal risk factors for neglecting the use of self-management strategies, e.g. a lack of energy or confusion through the many choices of strategies [14, 29].

Current results indicate that we need to adopt a broader perspective on coping with depression that exceeds beyond merely referring to self-management strategies [2]. This means that self-management entails more than specific strategies, i.e. day-to-day tasks an individual undertakes to control or reduce the impact of the condition [2, 11, 38]. In fact, a broader approach to self-management is expected to interact with introspection and empowerment, and thus the evolvement of experiential knowledge.

The proposition for a broad perspective on coping with depression is echoed in the recovery approach [24]. This method suggests that engaging in self-management of a mental disorder needs a holistic view. A holistic approach is characterised by treating the entire person, taking into account the physical, mental, and social factors and needs of a particular patient, not solely focusing on symptoms and diagnoses [8, 21]. Thus, personal aspects as well as factors in the wider environment are considered relevant to cope with the disorder [5, 24, 36]. Treatments for severe psychiatric disorders are increasingly based on the principles of the recovery approach. However, little attention has been paid to this approach in mental health care for depression. In line with the study of Chambers et al. [11] the current research acknowledges the importance and accuracy of the recovery approach in mental health care for depression. Chambers et al. [11] identified facilitating factors for self-management, suggesting ‘powerful agents’ such as hope, confidence and motivation that could help to manage depression. These ‘powerful agents’, as well as a greater emphasis on autonomy and a holistic approach in mental health care, reflect the results of the current study.

### Strengths and limitations

A strength of the study is the qualitative design, which allowed participants to give an in-depth description of their experiences and encouraged a holistic perspective on the dynamics of coping with depression. This led to a more inclusive understanding of the complexities of long-term coping with depression. Moreover, involving many stakeholders throughout the research process strengthened the reliability of the results, and allowed to validate the proposed model (Fig. 1) as a fruitful starting point for further research. The current findings overlap with experiential knowledge in other chronic mental illnesses, such as trauma exposure and psychoses. This suggests universal applicable principles in coping with a mental disorder.

However, this research is a first exploration of experiential knowledge in depression. Due to this limitation, the data does not allow to draw definite conclusions about the development of experiential knowledge and its relation to deploying self-management strategies. Furthermore, the back translation of the evolvement of experiential knowledge to individual patients is complex and requires balancing between generalizability of results and the uniqueness of each patient. The small sample size and heterogeneity of patients with chronic and/or recurrent depression makes it difficult to explain the influence of clinical and demographic details on the development of experiential knowledge. Specifically, the generalizability of the findings is limited because the majority of participants were Dutch and highly-educated. Moreover, all participants engaged in mental health care. Participants suggested a different information need and other coping styles in patients with chronic and/or recurrent depression, because of acquired competences in mental health therapies and previous experience in coping with depression. This limits the generalizability of findings to a group of depressive patients with a single depressive episode or starting down a path of recovery. Taken together, more specific research is needed to obtain a deeper understanding of individual clinical details affecting the development of experiential knowledge and deployment of self-management strategies.

Hypotheses can be derived from this study, which lays the groundwork for future research into the evolvement of experiential knowledge and the deployment of self-management strategies in depression. A bigger sample with a wider scope can be used to validate and develop the model of experiential knowledge. To specify the exact course of the development of these concepts, future research can address the following question: *What is needed to benefit from self-management strategies and experiential knowledge on the long-term when suffering from depression?* Including depressive patients who are not successful in the use of self-management can help to fill this knowledge gap. The role of mental health care in facilitating the pathway to experiential knowledge should also be addressed. Furthermore, the use of medication and the course of the depression (chronic, single or recurrent depression) may influence feelings of empowerment and introspection, and thus the evolvement of experiential knowledge and self-management strategies. Therefore, these topics should be discussed in future research on experiential knowledge in depression.

## Conclusion

In conclusion, the present study shows that the evolvement of experiential knowledge and the deployment of self-management strategies is a complex cyclical process. The proposed holistic approach towards the relation between the development of experiential knowledge and sustainable deployment of self-management strategies provides a promising perspective on long-term coping with depression, both in research, and in practice.

## Supplementary information

**Additional file 1.** Telephone screening.

**Additional file 2.** Sheehan et al. [42] MINI International Psychiatric Interview for DSM-IV-TR 5.0.0.

**Additional file 3.** Interview guide.

## Data Availability

All our study-related information is stored in secure folders with limited access. Electronic data files are stored on a file system with access restricted to designated researchers and data managers. The dataset is available from the corresponding author at Pro Persona Mental Health Care.

## Abbreviations

MINI interview: Mini International Neuropsychiatric Interview for DSM-IV-TR

## References

[1] Bal M (1997). Narratology: introduction to the theory of narrative. Toronto, Buffalo.

[2] Barlow J, Wright C, Sheasby J, Turner A, Hainsworth J (2002). Self-management approaches for people with chronic conditions: a review. Patient Educ Couns.

[3] Blume S (2017). In search of experiential knowledge. Innovation: Eur J Soc Sci Res.

[4] Boeije H (2009). Analysis in qualitative research: Sage publications.

[5] Boevink (2012). TREE: Towards recovery, empowerment and experiential expertise of users of psychiatric services. Empowerment, lifelong learning and recovery in mental health: towards a new paradigm.

[6] Boevink (2017). HEE!: Over Herstel, Empowerment en Ervaringsdeskundigheid in de psychiatrie: Maastricht University.

[7] Boevink W, Kroon H, van Vugt M, Delespaul P, van Os J (2016). A user-developed, user run recovery programme for people with severe mental illness: a randomised control trial. Psychosis.

[8] Bonney S, Stickley T (2008). Recovery and mental health: a review of the British literature. J Psychiatr Ment Health Nurs.

[9] Burda MHF, van den Akker M, van der Horst F, Lemmens P, Knottnerus JA (2016). Collecting and validating experiential expertise is doable but poses methodological challenges. J Clin Epidemiol.

[10] Burda MHF, van der Horst F, van den Akker M, Stork ADM, Crebolder H, van Attekum T (2012). et al. Identifying experiential expertise to support people with diabetes mellitus in applying for and participating effectively in paid work: a qualitative study. J Occup Environ Med.

[11] Chambers E, Cook S, Thake A, Foster A, Shaw S, Hutten R (2015). The self-management of longer-term depression: learning from the patient, a qualitative study. BMC Psychiatry.

[12] Clark NM, Becker MH, Janz NK, Lorig K, Rakowski W, Anderson L (1991). Self-management of chronic disease by older adults: a review and questions for research. J Aging Health.

[13] De Ridder D, Geenen R, Kuijer R, van Middendorp H (2008). Psychological adjustment to chronic disease. Lancet.

[14] DiMatteo MR, Lepper HS, Croghan TW (2000). Depression is a risk factor for noncompliance with medical treatment: meta-analysis of the effects of anxiety and depression on patient adherence. Arch Intern Med.

[15] Fox J. The role of the expert patient in the management of chronic illness. Br J Nurs. 2005;14(1):25–8. 10.12968/bjon.2005.14.1.17368 15750485

[16] Gilmer WS, Trivedi MH, Rush AJ, Wisniewski SR, Luther J, Howland RH (2005). Factors associated with chronic depressive episodes: a preliminary report from the STAR-D project. Acta Psychiatr Scand.

[17] Greden JF (2001). The burden of disease for treatment-resistant depression. J Clin Psychiatry.

[18] Greenhalgh T (2009). Chronic illness: beyond the expert patient. BMJ: British Medical Journal.

[19] Hardeveld F, Spijker J, De Graaf R, Nolen WA, Beekman ATF (2010). Prevalence and predictors of recurrence of major depressive disorder in the adult population. Acta Psychiatr Scand.

[20] Houle J, Gascon-Depatie M, Bélanger-Dumontier G, Cardinal C (2013). Depression self-management support: a systematic review. Patient Educ Couns.

[21] Jacob KS (2015). Recovery model of mental illness: a complementary approach to psychiatric care. Indian J Psychol Med.

[22] Johnson S, Lamb D, Marston L, Osborn D, Mason O, Henderson C (2018). Peer-supported self-management for people discharged from a mental health crisis team: a randomised controlled trial. Lancet.

[23] Keitner GI, Ryan CE, Solomon DA (2006). Realistic expectations and a disease management model for depressed patients with persistent symptoms. J Clin Psychiatry.

[24] Leamy M, Bird V, Le Boutillier C, Williams J, Slade M (2011). Conceptual framework for personal recovery in mental health: systematic review and narrative synthesis. Br J Psychiatry.

[25] Lecrubier Y, Sheehan DV, Weiller E, Amorim P, Bonora I, Sheehan KH (1997). The MINI international neuropsychiatric interview (MINI). A short diagnostic structured interview: reliability and validity according to the CIDI. Eur Psychiatry.

[26] Lloyd-Evans B, Mayo-Wilson E, Harrison B, Istead H, Brown E, Pilling S (2014). A systematic review and meta-analysis of randomised controlled trials of peer support for people with severe mental illness. BMC Psychiatry.

[27] Ludman EJ, Simon GE, Grothaus LC, Richards JE, Whiteside U, Stewart C (2015). Organized self-management support services for chronic depressive symptoms: a randomized controlled trial. Psychiatr Serv.

[28] Lustman PJ, Freedland KE, Griffith LS, Clouse RE (2000). Fluoxetine for depression in diabetes: a randomized double-blind placebo-controlled trial. Diabetes Care.

[29] Morgan AJ, Jorm AF (2009). Self-help strategies that are helpful for sub-threshold depression: a Delphi consensus study. J Affect Disord.

[30] Moussavi S, Chatterji S, Verdes E, Tandon A, Patel V, Ustun B (2007). Depression, chronic diseases, and decrements in health: results from the world health surveys. Lancet.

[31] Nemeroff CB (2007). Prevalence and management of treatment-resistant depression. J Clin Psychiatry.

[32] Overbeek I, Schruers K, Griez E (1999). Mini international neuropsychiatric interview: Nederlandse versie 5.0. 0. DSM-IV [Dutch version].

[33] Penninx BWJH, Nolen WA, Lamers F, Zitman FG, Smit JH, Spinhoven P (2011). Two-year course of depressive and anxiety disorders: results from the Netherlands study of depression and anxiety (NESDA). J Affect Disord.

[34] Richards D (2011). Prevalence and clinical course of depression: a review. Clin Psychol Rev.

[35] Sheehan DV, Janavs J, Baker R, Sheehan KH, Knapp E, Sheehan M. The Mini-International Neuropsychiatric Interview (MINI) English Version 5.0. 0. DSM-IV. Tampa: University of South Florida. 2006.

[36] Slade M (2009). Personal recovery and mental illness: A guide for mental health professionals: Cambridge University Press.

[37] Slomic M, Christiansen B, Soberg HL, Sveen U (2016). User involvement and experiential knowledge in interprofessional rehabilitation: a grounded theory study. BMC Health Serv Res.

[38] Van der Schaaf PS, Oderwald AK. Chronisch zieken over ervaringsdeskundigheid: een empirisch onderzoek naar de opvattingen over het begrip ervaringsdeskundigheid van chronisch zieken. In: Chronically ill people on experiential expertise: an empirical study about beliefs of chronically ill people concerning the concept of experiential expertise. Amsterdam: Vrije Universiteit van Amsterdam, Department of Metamedica; 1999.

[39] Van der Stel JC (2015). Functional recovery and self-regulation: assignments for both clients and psychiatrists. Tijdschrift voor Psychiatrie.

[40] Van Grieken RA, Kirkenier ACE, Koeter MWJ, Schene AH. Helpful self-management strategies to cope with enduring depression from the patients’ point of view: a concept map study. BMC Psychiatry. 2014;14(1):331. 10.1186/s12888-014-0331-7 PMC4272551 25495848

[41] Villaggi B, Provencher H, Coulombe S, Meunier S, Radziszewski S, Hudon C (2015). Self-management strategies in recovery from mood and anxiety disorders. Glob Qual Nurs Res.

[42] Yeung A, Feldman G, Fava M (2009). Self-management of depression: a manual for mental health and primary care professionals: Cambridge University Press.

