# Supplementary material for: An exploration of the conditions for deploying self-management strategies: a qualitative study of experiential knowledge in depression
Source: BMC Psychiatry. 2020 May 11;20:210. doi: 10.1186/s12888-020-02559-3 (PMC7212680; doi:10.1186/s12888-020-02559-3)
Supplement: Supplementary file 1 — Additional file 1. Telephone screening. [file 12888_2020_2559_MOESM1_ESM.docx]

| ***Date of screening:*** | | |
| --- | --- | --- |
| ***Introduction*** | | |
| - *Introducing yourself: junior researcher at Pro Persona Research & Radboud University Nijmegen* - *Explain the aim of the research ‘the strength of depression’* - *Explain the aim of the interview about experiential knowledge of depression* - *Explain the aim of this telephonic screening:*  1. *Inventory of the characteristics of the research sample* 2. *Personal questions about the course of suffering from depression* 3. *Transformation into anonymous data* 4. *Carefully handling this confidential information*  - *Explain the aim of the MINI-interview*  1. *Checking mental health issues, inclusion- and exclusion criteria* 2. *Structured interview*  - *Questions?* | | |
| ***Personal details*** | | |
| What is your date of birth? |  | |
| What is your cultural background? |  | |
| Country of birth: |  | |
| Country of birth mother: |  | |
| Country of birth father: |  | |
| Educational level (completed) |  | |
| Gender |  | |
| ***Details about the course of depression*** | | |
| In what year did you experience your first depressive episode? | |  |
| How many depressive episodes did you experience? | |  |
| When did you experience your last episode? | |  |
| Do you suffer from depression at the moment? | |  |
| Did you receive treatment for mental health issues? | |  |
| Are you currently in therapy?  If so: What kind of therapy do you receive? | |  |
| Do you currently use medication? | |  |
| ***Conducting MINI interview*** (Sheehan et al., 2006) | | |
| *See additional file 3 for the full version of the Mini International Psychiatric Interview for DSM-IV-TR (standardised clinical diagnostic interview)* Reference: *Sheehan, D. V., Janavs, J., Baker, R., Sheehan, K. H., Knapp, E., & Sheehan, M. (2006). The Mini-International Neuropsychiatric Interview (MINI) English Version 5.0. 0. DSM-IV. Tampa, FL: University of South Florida.* | | |

| ***Completion of telephone screening***  Thank you for your answers.  *Does participant meet the inclusion- and exclusion criteria?*   - *No: explain why and thank respondent for participating this far* - *Maybe: explain why, discuss with colleagues and call back later* - *Yes: schedule an interview with respondent* |
| --- |
