# Supplementary material for: An exploration of the conditions for deploying self-management strategies: a qualitative study of experiential knowledge in depression
Source: BMC Psychiatry. 2020 May 11;20:210. doi: 10.1186/s12888-020-02559-3 (PMC7212680; doi:10.1186/s12888-020-02559-3)
Supplement: Supplementary file 2 — Additional file 2. Sheehan et al. [42] MINI International Psychiatric Interview for DSM-IV-TR 5.0.0. [file 12888_2020_2559_MOESM2_ESM.pdf]

M.I.N.I.

MINI INTERNATIONAL NEUROPSYCHIATRIC INTERVIEW

English Version 5.0.0

DSM-IV

USA: **D. Sheehan, J. Janavs, R. Baker, K. Harnett-Sheehan, E. Knapp, M. Sheehan**
University of South Florida - Tampa

FRANCE: **Y. Lecrubier, E. Weiller, T. Hergueta, P. Amorim, L. I. Bonora, J. P. Lépine**
Hôpital de la Salpétrière - Paris

© Copyright 1992-2006 Sheehan DV & Lecrubier Y

All rights reserved. No part of this document may be reproduced or transmitted in any form, or by any means, electronic or mechanical, including photocopying, or by any information storage or retrieval system, without permission in writing from Dr. Sheehan or Dr. Lecrubier. Researchers and clinicians working in nonprofit or publicly owned settings (including universities, nonprofit hospitals, and government institutions) may make copies of a M.I.N.I. instrument for their own clinical and research use.

DISCLAIMER

Our aim is to assist in the assessment and tracking of patients with greater efficiency and accuracy. Before action is taken on any data collected and processed by this program, it should be reviewed and interpreted by a licensed clinician.

This program is not designed or intended to be used in the place of a full medical and psychiatric evaluation by a qualified licensed physician – psychiatrist. It is intended only as a tool to facilitate accurate data collection and processing of symptoms elicited by trained personnel.

M.I.N.I. 5.0.0 (July 1, 2006)

Patient Name:	________________	***Patient Number:***	________________
Date of Birth:	________________	***Time Interview Began:***	________________
Interviewer's Name:	________________	***Time Interview Ended:***	________________
Date of Interview:	________________	***Total Time:***	________________

	**MODULES**	**TIME FRAME**	**MEETS CRITERIA**	**DSM-IV**	**ICD-10**	
A	MAJOR DEPRESSIVE EPISODE	Current (2 weeks)	☐	296.20-296.26 Single	F32.x	☐
		Recurrent	☐	296.30-296.36 Recurrent	F33.x	☐
	MDE WITH MELANCHOLIC FEATURES	Current (2 weeks)	☐	296.20-296.26 Single	F32.x	☐
	Optional			296.30-296.36 Recurrent	F33.x	☐
B	DYSTHYMIA	Current (Past 2 years)	☐	300.4	F34.1	☐
C	SUICIDALITY	Current (Past Month)<br>Risk: ☐ Low ☐ Medium ☐ High	☐			☐
D	MANIC EPISODE	Current	☐	296.00-296.06	F30.x-F31.9	☐
		Past	☐			
	HYPOMANIC EPISODE	Current	☐	296.80-296.89	F31.8-F31.9/F34.0	☐
		Past	☐			
E	PANIC DISORDER	Current (Past Month)	☐	300.01/300.21	F40.01-F41.0	☐
		Lifetime	☐			
F	AGORAPHOBIA	Current	☐	300.22	F40.00	☐
G	SOCIAL PHOBIA (Social Anxiety Disorder)	Current (Past Month)	☐	300.23	F40.1	☐
H	OBSESSIVE-COMPULSIVE DISORDER	Current (Past Month)	☐	300.3	F42.8	☐
I	POSTTRAUMATIC STRESS DISORDER	Current (Past Month)	☐	309.81	F43.1	☐
J	ALCOHOL DEPENDENCE	Past 12 Months	☐	303.9	F10.2x	☐
	ALCOHOL ABUSE	Past 12 Months	☐	305.00	F10.1	☐
K	SUBSTANCE DEPENDENCE (Non-alcohol)	Past 12 Months	☐	304.00-.90/305.20-.90	F11.1-F19.1	☐
	SUBSTANCE ABUSE (Non-alcohol)	Past 12 Months	☐	304.00-.90/305.20-.90	F11.1-F19.1	☐
L	PSYCHOTIC DISORDERS	Lifetime	☐	295.10-295.90/297.1/	F20.xx-F29	☐
		Current	☐	297.3/293.81/293.82/<br>293.89/298.8/298.9		
	MOOD DISORDER WITH PSYCHOTIC FEATURES	Lifetime	☐	296.24/296.34/296.44	F32.3/F33.3/ ☐	
		Current	☐	296.24/296.34/296.44	F30.2/F31.2/F31.5<br>F31.8/F31.9/F39 ☐	
M	ANOREXIA NERVOSA	Current (Past 3 Months)	☐	307.1	F50.0	☐
N	BULIMIA NERVOSA	Current (Past 3 Months)	☐	307.51	F50.2	☐
	ANOREXIA NERVOSA, BINGE EATING/PURGING TYPE	Current	☐	307.1	F50.0	☐

O	GENERALIZED ANXIETY DISORDER	Current (Past 6 Months)	☐	300.02	F41.1	☐
P	ANTISOCIAL PERSONALITY DISORDER<br>Optional	Lifetime	☐	301.7	F60.2	☐

Which problem troubles you the most? Indicate your response by checking the appropriate check box(es).

GENERAL INSTRUCTIONS

The M.I.N.I. was designed as a brief structured interview for the major Axis I psychiatric disorders in DSM-IV and ICD-10. Validation and reliability studies have been done comparing the M.I.N.I. to the SCID-P for DSM-III-R and the CIDI (a structured interview developed by the World Health Organization for lay interviewers for ICD-10). The results of these studies show that the M.I.N.I. has acceptably high validation and reliability scores, but can be administered in a much shorter period of time (mean 18.7 ± 11.6 minutes, median 15 minutes) than the above referenced instruments. It can be used by clinicians, after a brief training session. Lay interviewers require more extensive training.

INTERVIEW:

In order to keep the interview as brief as possible, inform the patient that you will conduct a clinical interview that is more structured than usual, with very precise questions about psychological problems which require a yes or no answer.

GENERAL FORMAT:

The M.I.N.I. is divided into **modules** identified by letters, each corresponding to a diagnostic category.
•At the beginning of each diagnostic module (except for psychotic disorders module), screening question(s) corresponding to the main criteria of the disorder are presented in a **gray box**.
•At the end of each module, diagnostic box(es) permit the clinician to indicate whether diagnostic criteria are met.

CONVENTIONS:

Sentences written in « normal font » should be read exactly as written to the patient in order to standardize the assessment of diagnostic criteria.

Sentences written in « CAPITALS » should not be read to the patient. They are instructions for the interviewer to assist in the scoring of the diagnostic algorithms.

Sentences written in « **bold** » indicate the time frame being investigated. The interviewer should read them as often as necessary. Only symptoms occurring during the time frame indicated should be considered in scoring the responses.

Answers with an arrow above them (➨) indicate that one of the criteria necessary for the diagnosis(es) is not met. In this case, the interviewer should go to the end of the module, circle « **NO** » in all the diagnostic boxes and move to the next module.

When terms are separated by a *slash (/)* the interviewer should read only those symptoms known to be present in the patient (for example, question H6).

Phrases in (parentheses) are clinical examples of the symptom. These may be read to the patient to clarify the question.

RATING INSTRUCTIONS:

All questions must be rated. The rating is done at the right of each question by circling either Yes or No. Clinical judgment by the rater should be used in coding the responses. The rater should ask for examples when necessary, to ensure accurate coding. The patient should be encouraged to ask for clarification on any question that is not absolutely clear.

The clinician should be sure that <u>each dimension</u> of the question is taken into account by the patient (for example, time frame, frequency, severity, and/or alternatives).
<u>Symptoms better accounted for by an organic cause or by the use of alcohol or drugs should not be coded positive in the M.I.N.I.</u> The M.I.N.I. Plus has questions that investigate these issues.

For any questions, suggestions, need for a training session, or information about updates of the M.I.N.I., please contact :

David V Sheehan, M.D., M.B.A.
University of South Florida College of Medicine
3515 East Fletcher Avenue
Tampa, FL USA 33613-4788
tel : +1 813 974 4544; fax : +1 813 974 4575
e-mail : dsheehan@hsc.usf.edu

Yves Lecrubier, M.D. / Thierry Hergueta, M.S.
INSERM U302
Hôpital de la Salpétrière
47, boulevard de l'Hôpital
F. 75651 PARIS, FRANCE
tel : +33 (0) 1 42 16 16 59; fax : +33 (0) 1 45 85 28 00
e-mail : hergueta@ext.jussieu.fr

A. MAJOR DEPRESSIVE EPISODE

(➡ MEANS : GO TO THE DIAGNOSTIC BOXES, CIRCLE NO IN ALL DIAGNOSTIC BOXES, AND MOVE TO THE NEXT MODULE)

A1		Have you been consistently depressed or down, most of the day, nearly every day, for the past two weeks?	NO	YES
A2		In the past two weeks, have you been much less interested in most things or much less able to enjoy the things you used to enjoy most of the time?	NO	YES
		IS **A1** OR **A2** CODED **YES?**	➡ NO	YES
A3		**Over the past two weeks, when you felt depressed or uninterested:**		
	a	Was your appetite decreased or increased nearly every day? Did your weight decrease or increase without trying intentionally (i.e., by ±5% of body weight or ±8 lbs. or ±3.5 kgs., for a 160 lb./70 kg. person in a month)? IF **YES** TO EITHER, CODE **YES**.	NO	YES *
	b	Did you have trouble sleeping nearly every night (difficulty falling asleep, waking up in the middle of the night, early morning wakening or sleeping excessively)?	NO	YES
	c	Did you talk or move more slowly than normal or were you fidgety, restless or having trouble sitting still almost every day?	NO	YES *
	d	Did you feel tired or without energy almost every day?	NO	YES
	e	Did you feel worthless or guilty almost every day?	NO	YES
	f	Did you have difficulty concentrating or making decisions almost every day?	NO	YES
	g	Did you repeatedly consider hurting yourself, feel suicidal, or wish that you were dead?	NO	YES

ARE **5** OR MORE ANSWERS (**A1-A3**) CODED **YES**?

NO **YES ***

MAJOR DEPRESSIVE EPISODE, CURRENT

IF PATIENT HAS CURRENT MAJOR DEPRESSIVE EPISODE CONTINUE TO A4, OTHERWISE MOVE TO MODULE B:

A4	a	During your lifetime, did you have other episodes of two weeks or more when you felt depressed or uninterested in most things, and had most of the problems we just talked about?	➡ NO	YES
	b	In between 2 episodes of depression, did you ever have an interval of at least 2 months, without any depression and any loss of interest?		

NO **YES**

MAJOR DEPRESSIVE EPISODE, RECURRENT

* If patient has Major Depressive Episode, Current, use this information in coding the corresponding questions on page 5 (A6d, A6e).

MAJOR DEPRESSIVE EPISODE WITH MELANCHOLIC FEATURES (optional)

(➡ MEANS : GO TO THE DIAGNOSTIC BOX, CIRCLE NO, AND MOVE TO THE NEXT MODULE)

IF THE PATIENT CODES POSITIVE FOR A CURRENT MAJOR DEPRESSIVE EPISODE (**A3** = **YES**), EXPLORE THE FOLLOWING:

A5	a	During the most severe period of the current depressive episode, did you lose almost completely your ability to enjoy nearly everything?	NO	YES
	b	During the most severe period of the current depressive episode, did you lose your ability to respond to things that previously gave you pleasure, or cheered you up?<br>**IF NO:** When something good happens does it fail to make you feel better, even temporarily?	NO	YES
		IS EITHER **A5a** OR **A5b** CODED **YES**?	➡<br>NO	YES
A6		**Over the past two week period, when you felt depressed and uninterested:**		
	a	Did you feel depressed in a way that is different from the kind of feeling you experience when someone close to you dies?	NO	YES
	b	Did you feel regularly worse in the morning, almost every day?	NO	YES
	c	Did you wake up at least 2 hours before the usual time of awakening and have difficulty getting back to sleep, almost every day?	NO	YES
	d	IS **A3c** CODED **YES** (PSYCHOMOTOR RETARDATION OR AGITATION)?	NO	YES
	e	IS **A3a** CODED **YES** FOR ANOREXIA OR WEIGHT LOSS?	NO	YES
	f	Did you feel excessive guilt or guilt out of proportion to the reality of the situation?	NO	YES

ARE **3** OR MORE **A6** ANSWERS CODED **YES**?

NO **YES**

Major Depressive Episode
with
Melancholic Features
Current

B. DYSTHYMIA

(➡ MEANS : GO TO THE DIAGNOSTIC BOX, CIRCLE NO, AND MOVE TO THE NEXT MODULE)

IF PATIENT'S SYMPTOMS CURRENTLY MEET CRITERIA FOR MAJOR DEPRESSIVE EPISODE, DO NOT EXPLORE THIS MODULE.

B1		Have you felt sad, low or depressed most of the time for the last two years?	➡ NO	YES
B2		Was this period interrupted by your feeling OK for two months or more?	NO	➡ YES
B3		**During this period of feeling depressed most of the time:**		
	a	Did your appetite change significantly?	NO	YES
	b	Did you have trouble sleeping or sleep excessively?	NO	YES
	c	Did you feel tired or without energy?	NO	YES
	d	Did you lose your self-confidence?	NO	YES
	e	Did you have trouble concentrating or making decisions?	NO	YES
	f	Did you feel hopeless?	NO	YES
		ARE **2** OR MORE **B3** ANSWERS CODED **YES**?	➡ NO	YES
B4		Did the symptoms of depression cause you significant distress or impair your ability to function at work, socially, or in some other important way?		

NO **YES**

DYSTHYMIA
CURRENT

C. SUICIDALITY

In the past month did you:

		NO	YES	Points
C1	Suffer any accident?	NO	YES	0
	IF NO TO C1, SKIP TO C2; IF YES, ASK C1a,:			
C1a	Plan or intend to hurt yourself in that accident either passively or actively?	NO	YES	0
	IF NO TO C1a, SKIP TO C2: IF YES, ASK C1b,:			
C1b	Did you intend to die as a result of this accident?	NO	YES	0
C2	Think that you would be better off dead or wish you were dead?	NO	YES	1
C3	Want to harm yourself or to hurt or to injure yourself?	NO	YES	2
C4	Think about suicide?	NO	YES	6

IF YES, ASK ABOUT THE INTENSITY AND FREQUENCY OF THE SUICIDAL IDEATION:

Frequency		Intensity	
Occasionally	❒	Mild	❒
Often	❒	Moderate	❒
Very often	❒	Severe	❒

→ Can you control these impulses and state that you will not act on them while in this program?
Only score 8 points if response is NO. NO YES 8

		NO	YES	Points
C5	Have a suicide plan?	NO	YES	8
C6	Take any active steps to prepare to injure yourself or to prepare for a suicide attempt in which you expected or intended to die?	NO	YES	9
C7	Deliberately injure yourself without intending to kill yourself?	NO	YES	4
C8	Attempt suicide?	NO	YES	10

Hoped to be rescued / survive ❒
Expected / intended to die ❒

In your lifetime:

		NO	YES	Points
C9	Did you ever make a suicide attempt?	NO	YES	4

IS AT LEAST **1** OF THE ABOVE (EXCEPT C1) CODED **YES**?

IF YES, ADD THE TOTAL NUMBER OF POINTS FOR THE ANSWERS (C1-C9) CHECKED 'YES' AND SPECIFY THE LEVEL OF SUICIDE RISK AS INDICATED IN THE DIAGNOSTIC BOX:

MAKE ANY ADDITIONAL COMMENTS ABOUT YOUR ASSESSMENT OF THIS PATIENT'S CURRENT AND NEAR FUTURE SUICIDE RISK IN THE SPACE BELOW:

NO **YES**

SUICIDE RISK CURRENT

1-8 points	Low	❒
9-16 points	Moderate	❒
$\geq$ 17 points	High	❒

D. (HYPO) MANIC EPISODE

(➡ MEANS : GO TO THE DIAGNOSTIC BOXES, CIRCLE NO IN ALL DIAGNOSTIC BOXES, AND MOVE TO THE NEXT MODULE)

D1	a	Have you **ever** had a period of time when you were feeling 'up' or 'high' or 'hyper' or so full of energy or full of yourself that you got into trouble, or that other people thought you were not your usual self? (Do not consider times when you were intoxicated on drugs or alcohol.)<br><br>IF PATIENT IS PUZZLED OR UNCLEAR ABOUT WHAT YOU MEAN BY 'UP' OR 'HIGH' OR 'HYPER', CLARIFY AS FOLLOWS: By 'up' or 'high' or 'hyper' I mean: having elated mood; increased energy; needing less sleep; having rapid thoughts; being full of ideas; having an increase in productivity, motivation, creativity, or impulsive behavior.<br><br>IF NO, CODE NO TO **D1b**: IF **YES** ASK:	NO	YES
	b	Are you currently feeling 'up' or 'high' or 'hyper' or full of energy?	NO	YES
D2	a	Have you **ever** been persistently irritable, for several days, so that you had arguments or verbal or physical fights, or shouted at people outside your family? Have you or others noticed that you have been more irritable or over reacted, compared to other people, even in situations that you felt were justified?<br><br>IF NO, CODE NO TO **D2b**: IF **YES** ASK:	NO	YES
	b	Are you currently feeling persistently irritable?	NO	YES
		IS **D1a** OR **D2a** CODED **YES**?	➡<br>NO	YES

D3 IF **D1b** OR **D2b** = **YES**: EXPLORE THE **CURRENT** AND THE MOST SYMPTOMATIC **PAST** EPISODE, OTHERWISE IF **D1b** AND **D2b** = **NO**: EXPLORE ONLY THE MOST SYMPTOMATIC **PAST** EPISODE

During the times when you felt high, full of energy, or irritable did you:

		Current Episode		Past Episode	
a	Feel that you could do things others couldn't do, or that you were an especially important person?<br>IF **YES**, ASK FOR EXAMPLES.<br>THE EXAMPLES ARE CONSISTENT WITH A DELUSIONAL IDEA. ❐ No ❐ Yes	NO	YES	NO	YES
b	Need less sleep (for example, feel rested after only a few hours sleep)?	NO	YES	NO	YES
c	Talk too much without stopping, or so fast that people had difficulty understanding?	NO	YES	NO	YES
d	Have racing thoughts?	NO	YES	NO	YES
e	Become easily distracted so that any little interruption could distract you?	NO	YES	NO	YES
f	Become so active or physically restless that others were worried about you?	NO	YES	NO	YES
g	Want so much to engage in pleasurable activities that you ignored the risks or consequences (for example, spending sprees, reckless driving, or sexual indiscretions)?	NO	YES	NO	YES

	Current Episode		Past Episode	
**D3 (SUMMARY):** ARE **3** OR MORE **D3** ANSWERS CODED **YES** (OR **4** OR MORE IF **D1a** IS **NO** (IN RATING PAST EPISODE) AND **D1b** IS **NO (**IN RATING CURRENT EPISODE)? RULE: ELATION/EXPANSIVENESS REQUIRES ONLY THREE D3 SYMPTOMS WHILE IRRITABLE MOOD ALONE REQUIRES **4** OF THE **D3** SYMPTOMS. VERIFY IF THE SYMPTOMS OCCURRED DURING THE SAME TIME PERIOD.	NO	YES	➡ NO	YES
D4 Did these symptoms last at least a week **and** cause significant problems at home, at work, socially, or at school, **or** were you hospitalized for these problems?	NO ↓	YES ↓	NO ↓	YES ↓
THE EPISODE EXPLORED WAS A:	❒ ***HYPOMANIC EPISODE***	❒ ***MANIC EPISODE***	❒ ***HYPOMANIC EPISODE***	❒ ***MANIC EPISODE***

IS **D4** CODED **NO**?

SPECIFY IF THE EPISODE IS CURRENT OR PAST.

NO **YES**

HYPOMANIC EPISODE

CURRENT ❒
PAST ❒

IS **D4** CODED **YES**?

SPECIFY IF THE EPISODE IS CURRENT OR PAST.

NO **YES**

MANIC EPISODE

CURRENT ❒
PAST ❒

E. PANIC DISORDER

(➡ MEANS : CIRCLE NO IN E5, E6 AND E7 AND SKIP TO F1)

E1	a	Have you, on more than one occasion, had spells or attacks when you **suddenly** felt anxious, frightened, uncomfortable or uneasy, even in situations where most people would not feel that way?	➡ NO	YES
	b	Did the spells surge to a peak within 10 minutes of starting?	➡ NO	YES
E2		At any time in the past, did any of those spells or attacks come on unexpectedly or occur in an unpredictable or unprovoked manner?	➡ NO	YES
E3		Have you ever had one such attack followed by a month or more of persistent concern about having another attack, or worries about the consequences of the attack or did you make a significant change in your behavior because of the attacks (e.g., shopping only with a companion, not wanting to leave your house, visiting the emergency room repeatedly, or seeing your doctor more frequently because of the symptoms?	NO	YES
E4		**During the worst spell that you can remember:**		
	a	Did you have skipping, racing or pounding of your heart?	NO	YES
	b	Did you have sweating or clammy hands?	NO	YES
	c	Were you trembling or shaking?	NO	YES
	d	Did you have shortness of breath or difficulty breathing?	NO	YES
	e	Did you have a choking sensation or a lump in your throat?	NO	YES
	f	Did you have chest pain, pressure or discomfort?	NO	YES
	g	Did you have nausea, stomach problems or sudden diarrhea?	NO	YES
	h	Did you feel dizzy, unsteady, lightheaded or faint?	NO	YES
	i	Did things around you feel strange, unreal, detached or unfamiliar, or did you feel outside of or detached from part or all of your body?	NO	YES
	j	Did you fear that you were losing control or going crazy?	NO	YES
	k	Did you fear that you were dying?	NO	YES
	l	Did you have tingling or numbness in parts of your body?	NO	YES
	m	Did you have hot flushes or chills?	NO	YES
E5		ARE BOTH **E3,** AND **4** OR MORE **E4** ANSWERS, CODED **YES**?<br>IF YES TO E5, SKIP TO E7.	NO	YES<br>***PANIC DISORDER LIFETIME***
E6		IF **E5** = **NO,** ARE ANY E4 ANSWERS CODED **YES**?<br>THEN SKIP TO **F1.**	NO	YES<br>***LIMITED SYMPTOM ATTACKS LIFETIME***
E7		In the past month, did you have such attacks repeatedly (2 or more) followed by persistent concern about having another attack?	NO	YES<br>***PANIC DISORDER CURRENT***

F. AGORAPHOBIA

F1	Do you feel anxious or uneasy in places or situations where you might have a panic attack or the panic-like symptoms we just spoke about, or where help might not be available or escape might be difficult: like being in a crowd, standing in a line (queue), when you are alone away from home or alone at home, or when crossing a bridge, traveling in a bus, train or car?	NO YES

IF **F1** = **NO**, CIRCLE **NO** IN **F2**.

F2	Do you fear these situations so much that you avoid them, or suffer through them, or need a companion to face them?	NO YES<br>***AGORAPHOBIA CURRENT***

IS **F2** (CURRENT AGORAPHOBIA) CODED **NO**

and

IS **E7** (CURRENT PANIC DISORDER) CODED **YES**?

NO YES

PANIC DISORDER without Agoraphobia CURRENT

IS **F2** (CURRENT AGORAPHOBIA) CODED **YES**

and

IS **E7** (CURRENT PANIC DISORDER) CODED **YES**?

NO YES

PANIC DISORDER with Agoraphobia CURRENT

IS **F2** (CURRENT AGORAPHOBIA) CODED **YES**

and

IS **E5** (PANIC DISORDER LIFETIME) CODED **NO**?

NO YES

AGORAPHOBIA, CURRENT without history of Panic Disorder

G. SOCIAL PHOBIA (Social Anxiety Disorder)

(➡ MEANS : GO TO THE DIAGNOSTIC BOX, CIRCLE NO AND MOVE TO THE NEXT MODULE)

		➡	
G1	In the past month, were you fearful or embarrassed being watched, being the focus of attention, or fearful of being humiliated? This includes things like speaking in public, eating in public or with others, writing while someone watches, or being in social situations.	NO	YES
G2	Is this social fear excessive or unreasonable?	➡ NO	YES
G3	Do you fear these social situations so much that you avoid them or suffer through them?	➡ NO	YES

G4 Do these social fears disrupt your normal work or social functioning or cause you significant distress?

SUBTYPES

Do you fear and avoid 4 or more social situations?

If YES Generalized social phobia (social anxiety disorder)

If NO Non-generalized social phobia (social anxiety disorder)

NOTE TO INTERVIEWER: PLEASE ASSESS WHETHER THE SUBJECT'S FEARS ARE RESTRICTED TO NON-GENERALIZED ("ONLY 1 OR SEVERAL") SOCIAL SITUATIONS OR EXTEND TO GENERALIZED ("MOST") SOCIAL SITUATIONS. "MOST" SOCIAL SITUATIONS IS USUALLY OPERATIONALIZED TO MEAN 4 OR MORE SOCIAL SITUATIONS, ALTHOUGH THE DSM-IV DOES NOT EXPLICITLY STATE THIS.

EXAMPLES OF SUCH SOCIAL SITUATIONSTYPICALLY INCLUDE INITIATING OR MAINTAINING A CONVERSATION, PARTICIPATING IN SMALL GROUPS, DATING, SPEAKING TO AUTHORITY FIGURES, ATTENDING PARTIES, PUBLIC SPEAKING, EATING IN FRONT OF OTHERS, URINATING IN A PUBLIC WASHROOM, ETC.

NO **YES**

SOCIAL PHOBIA
(Social Anxiety Disorder)
CURRENT

GENERALIZED ❒

NON-GENERALIZED ❒

H. OBSESSIVE-COMPULSIVE DISORDER

(➡ MEANS: GO TO THE DIAGNOSTIC BOX, CIRCLE NO AND MOVE TO THE NEXT MODULE)

H1	In the past month, have you been bothered by recurrent thoughts, impulses, or images that were unwanted, distasteful, inappropriate, intrusive, or distressing? (For example, the idea that you were dirty, contaminated or had germs, **or** fear of contaminating others, **or** fear of harming someone even though you didn't want to, **or** fearing you would act on some impulse, **or** fear or superstitions that you would be responsible for things going wrong, **or** obsessions with sexual thoughts, images or impulses, **or** hoarding, collecting, **or** religious obsessions.)<br><br>(DO NOT INCLUDE SIMPLY EXCESSIVE WORRIES ABOUT REAL LIFE PROBLEMS. DO NOT INCLUDE OBSESSIONS DIRECTLY RELATED TO EATING DISORDERS, SEXUAL DEVIATIONS, PATHOLOGICAL GAMBLING, OR ALCOHOL OR DRUG ABUSE BECAUSE THE PATIENT MAY DERIVE PLEASURE FROM THE ACTIVITY AND MAY WANT TO RESIST IT ONLY BECAUSE OF ITS NEGATIVE CONSEQUENCES.)	NO YES<br>↓<br>SKIP TO **H4**
H2	Did they keep coming back into your mind even when you tried to ignore or get rid of them?	NO YES<br>↓<br>SKIP TO **H4**
H3	Do you think that these obsessions are the product of your own mind and that they are not imposed from the outside?	NO YES<br>**obsessions**
H4	In the past month, did you do something repeatedly without being able to resist doing it, like washing or cleaning excessively, counting or checking things over and over, or repeating, collecting, arranging things, or other superstitious rituals?	NO YES<br>**compulsions**
	IS **H3** OR **H4** CODED **YES**?	➡<br>NO YES
H5	Did you recognize that either these obsessive thoughts or these compulsive behaviors were excessive or unreasonable?	➡<br>NO YES
H6	Did these obsessive thoughts and/or compulsive behaviors significantly interfere with your normal routine, your work or school, your usual social activities, or relationships, or did they take more than one hour a day?	**NO YES**<br><br>***O.C.D. CURRENT***

I. POSTTRAUMATIC STRESS DISORDER (optional)

(➡ MEANS : GO TO THE DIAGNOSTIC BOX, CIRCLE NO, AND MOVE TO THE NEXT MODULE)

I1	Have you ever experienced or witnessed or had to deal with an extremely traumatic event that included actual or threatened death or serious injury to you or someone else?<br><br>EXAMPLES OF TRAUMATIC EVENTS INCLUDE: SERIOUS ACCIDENTS, SEXUAL OR PHYSICAL ASSAULT, A TERRORIST ATTACK, BEING HELD HOSTAGE, KIDNAPPING, FIRE, DISCOVERING A BODY, SUDDEN DEATH OF SOMEONE CLOSE TO YOU, WAR, OR NATURAL DISASTER.	➡ NO	YES
I2	Did you respond with intense fear, helplessness or horror?	➡ NO	YES
I3	During the past month, have you re-experienced the event in a distressing way (such as, dreams, intense recollections, flashbacks or physical reactions)?	➡ NO	YES
I4	**In the past month:**		
a	Have you avoided thinking about or talking about the event ?	NO	YES
b	Have you avoided activities, places or people that remind you of the event?	NO	YES
c	Have you had trouble recalling some important part of what happened?	NO	YES
d	Have you become much less interested in hobbies or social activities?	NO	YES
e	Have you felt detached or estranged from others?	NO	YES
f	Have you noticed that your feelings are numbed?	NO	YES
g	Have you felt that your life will be shortened or that you will die sooner than other people?	NO	YES
	ARE **3** OR MORE **I4** ANSWERS CODED **YES**?	➡ NO	YES
I5	**In the past month:**		
a	Have you had difficulty sleeping?	NO	YES
b	Were you especially irritable or did you have outbursts of anger?	NO	YES
c	Have you had difficulty concentrating?	NO	YES
d	Were you nervous or constantly on your guard?	NO	YES
e	Were you easily startled?	NO	YES
	ARE **2** OR MORE **I5** ANSWERS CODED **YES**?	➡ NO	YES
I6	During the past month, have these problems significantly interfered with your work or social activities, or caused significant distress?	**NO**	**YES**

NO **YES**

POSTTRAUMATIC STRESS DISORDER CURRENT

J. ALCOHOL ABUSE AND DEPENDENCE

(➡ MEANS: GO TO DIAGNOSTIC BOXES, CIRCLE **NO** IN BOTH AND MOVE TO THE NEXT MODULE)

		➡	
J1	**In the past 12 months**, have you had 3 or more alcoholic drinks within a 3 hour period on 3 or more occasions?	NO	YES

J2 **In the past 12 months**:

a	Did you need to drink more in order to get the same effect that you got when you first started drinking?	NO	YES
b	When you cut down on drinking did your hands shake, did you sweat or feel agitated? Did you drink to avoid these symptoms or to avoid being hungover, for example, "the shakes", sweating or agitation?<br>IF **YES** TO EITHER, CODE **YES**.	NO	YES
c	During the times when you drank alcohol, did you end up drinking more than you planned when you started?	NO	YES
d	Have you tried to reduce or stop drinking alcohol but failed?	NO	YES
e	On the days that you drank, did you spend substantial time in obtaining alcohol, drinking, or in recovering from the effects of alcohol?	NO	YES
f	Did you spend less time working, enjoying hobbies, or being with others because of your drinking?	NO	YES
g	Have you continued to drink even though you knew that the drinking caused you health or mental problems?	NO	YES

ARE **3** OR MORE **J2** ANSWERS CODED **YES**?

* IF YES, SKIP J3 QUESTIONS, CIRCLE N/A IN THE ABUSE BOX AND MOVE TO THE NEXT DISORDER. DEPENDENCE PREEMPTS ABUSE.

NO **YES***

ALCOHOL DEPENDENCE
CURRENT

J3 **In the past 12 months:**

a	Have you been intoxicated, high, or hungover more than once when you had other responsibilities at school, at work, or at home? Did this cause any problems?<br>(CODE **YES** ONLY IF THIS CAUSED PROBLEMS.)	NO	YES
b	Were you intoxicated more than once in any situation where you were physically at risk, for example, driving a car, riding a motorbike, using machinery, boating, etc.?	NO	YES
c	Did you have legal problems more than once because of your drinking, for example, an arrest or disorderly conduct?	NO	YES
d	Did you continue to drink even though your drinking caused problems with your family or other people?	NO	YES

ARE **1** OR MORE **J3** ANSWERS CODED **YES**?

NO **N/A** **YES**

ALCOHOL ABUSE
CURRENT

K. NON-ALCOHOL PSYCHOACTIVE SUBSTANCE USE DISORDERS

(➡ MEANS : GO TO THE DIAGNOSTIC BOXES, CIRCLE NO IN ALL DIAGNOSTIC BOXES, AND MOVE TO THE NEXT MODULE)

Now I am going to show you / read to you a list of street drugs or medicines.

			➡	
K1	a	**In the past 12 months**, did you take any of these drugs more than once, to get high, to feel better, or to change your mood?	NO	YES

CIRCLE EACH DRUG TAKEN:

Stimulants: amphetamines, "speed", crystal meth, “crank”, "rush", Dexedrine, Ritalin, diet pills.

Cocaine: snorting, IV, freebase, crack, "speedball".

Narcotics: heroin, morphine, Dilaudid, opium, Demerol, methadone, codeine, Percodan, Darvon, OxyContin.

Hallucinogens: LSD ("acid"), mescaline, peyote, PCP ("angel dust", "peace pill"), psilocybin, STP, "mushrooms", “ecstasy”, MDA, MDMA, or ketamine (“special K”).

Inhalants: "glue", ethyl chloride, “rush”, nitrous oxide ("laughing gas"), amyl or butyl nitrate ("poppers").

Marijuana: hashish ("hash"), THC, "pot", "grass", "weed", "reefer".

Tranquilizers: Quaalude, Seconal ("reds"), Valium, Xanax, Librium, Ativan, Dalmane, Halcion, barbiturates, Miltown, GHB, Roofinol, “Roofies”.

Miscellaneous: steroids, nonprescription sleep or diet pills. Any others?

SPECIFY MOST USED DRUG(S): ______________________________

	CHECK ONE BOX
ONLY ONE DRUG / DRUG CLASS HAS BEEN USED	☐
ONLY THE MOST USED DRUG CLASS IS INVESTIGATED.	☐
EACH DRUG CLASS USED IS EXAMINED SEPARATELY (PHOTOCOPY K2 AND K3 AS NEEDED)	☐

b SPECIFY WHICH DRUG/DRUG CLASS WILL BE EXPLORED IN THE INTERVIEW BELOW IF THERE IS CONCURRENT OR SEQUENTIAL POLYSUBSTANCE USE:______________________________

K2 **Considering your use of (NAME THE DRUG / DRUG CLASS SELECTED), in the past 12 months:**

	a	Have you found that you needed to use more (NAME OF DRUG / DRUG CLASS SELECTED) to get the same effect that you did when you first started taking it?	NO	YES
	b	When you reduced or stopped using (NAME OF DRUG / DRUG CLASS SELECTED), did you have withdrawal symptoms (aches, shaking, fever, weakness, diarrhea, nausea, sweating, heart pounding, difficulty sleeping, or feeling agitated, anxious, irritable, or depressed)? Did you use any drug(s) to keep yourself from getting sick (withdrawal symptoms) or so that you would feel better?<br><br>IF **YES** TO EITHER, CODE **YES**.	NO	YES
	c	Have you often found that when you used (NAME OF DRUG / DRUG CLASS SELECTED), you ended up taking more than you thought you would?	NO	YES
	d	Have you tried to reduce or stop taking (NAME OF DRUG / DRUG CLASS SELECTED) but failed?	NO	YES
	e	On the days that you used (NAME OF DRUG / DRUG CLASS SELECTED), did you spend substantial time (>2 HOURS), obtaining, using or in recovering from the drug, or thinking about the drug?	NO	YES

f Did you spend less time working, enjoying hobbies, or being with family or friends because of your drug use? NO YES

g Have you continued to use (NAME OF DRUG / DRUG CLASS SELECTED), even though it caused you health or mental problems? NO YES

ARE **3** OR MORE **K2** ANSWERS CODED **YES**?

SPECIFY DRUG(S): ____________________

* IF YES, SKIP K3 QUESTIONS, CIRCLE N/A IN THE ABUSE BOX FOR THIS SUBSTANCE AND MOVE TO THE NEXT DISORDER.
DEPENDENCE PREEMPTS ABUSE.

**NO**	**YES** *
SUBSTANCE DEPENDENCE **CURRENT**	

Considering your use of (NAME THE DRUG CLASS SELECTED), in the past 12 months:

K3 a Have you been intoxicated, high, or hungover from (NAME OF DRUG / DRUG CLASS SELECTED) more than once, when you had other responsibilities at school, at work, or at home? Did this cause any problem? NO YES

(CODE **YES** ONLY IF THIS CAUSED PROBLEMS.)

b Have you been high or intoxicated from (NAME OF DRUG / DRUG CLASS SELECTED) more than once in any situation where you were physically at risk (for example, driving a car, riding a motorbike, using machinery, boating, etc.)? NO YES

c Did you have legal problems more than once because of your drug use, for example, an arrest or disorderly conduct? NO YES

d Did you continue to use (NAME OF DRUG / DRUG CLASS SELECTED), even though it caused problems with your family or other people? NO YES

ARE **1** OR MORE **K3** ANSWERS CODED **YES**?

SPECIFY DRUG(S): ____________________

**NO**	**N/A**	**YES**
SUBSTANCE ABUSE **CURRENT**		

L. PSYCHOTIC DISORDERS AND MOOD DISORDER WITH PSYCHOTIC FEATURES

ASK FOR AN EXAMPLE OF EACH QUESTION ANSWERED POSITIVELY. CODE **YES** ONLY IF THE EXAMPLES CLEARLY SHOW A DISTORTION OF THOUGHT OR OF PERCEPTION OR IF THEY ARE NOT CULTURALLY APPROPRIATE. BEFORE CODING, INVESTIGATE WHETHER DELUSIONS QUALIFY AS "BIZARRE".

DELUSIONS ARE "BIZARRE" IF: CLEARLY IMPLAUSIBLE, ABSURD, NOT UNDERSTANDABLE, AND CANNOT DERIVE FROM ORDINARY LIFE EXPERIENCE.

HALLUCINATIONS ARE SCORED "BIZARRE" IF: A VOICE COMMENTS ON THE PERSON'S THOUGHTS OR BEHAVIOR, OR WHEN TWO OR MORE VOICES ARE CONVERSING WITH EACH OTHER.

					BIZARRE
		Now I am going to ask you about unusual experiences that some people have.			
L1	a	Have you ever believed that people were spying on you, or that someone was plotting against you, or trying to hurt you?<br>**NOTE**: ASK FOR EXAMPLES TO RULE OUT ACTUAL STALKING.	NO	YES	YES
	b	**IF YES OR YES BIZARRE:** do you currently believe these things?	NO	YES	YES<br>➡**L6**
L2	a	Have you ever believed that someone was reading your mind or could hear your thoughts, or that you could actually read someone's mind or hear what another person was thinking?	NO	YES	YES
	b	**IF YES OR YES BIZARRE:** do you currently believe these things?	NO	YES	YES<br>➡**L6**
L3	a	Have you ever believed that someone or some force outside of yourself put thoughts in your mind that were not your own, or made you act in a way that was not your usual self? Have you ever felt that you were possessed?<br>CLINICIAN: ASK FOR EXAMPLES AND DISCOUNT ANY THAT ARE NOT PSYCHOTIC.	NO	YES	YES
	b	**IF YES OR YES BIZARRE:** do you currently believe these things?	NO	YES	YES<br>➡**L6**
L4	a	Have you ever believed that you were being sent special messages through the TV, radio, or newspaper, or that a person you did not personally know was particularly interested in you?	NO	YES	YES
	b	**IF YES OR YES BIZARRE:** do you currently believe these things?	NO	YES	YES<br>➡**L6**
L5	a	Have your relatives or friends ever considered any of your beliefs strange or unusual?<br>INTERVIEWER**:** ASK FOR EXAMPLES. ONLY CODE **YES** IF THE EXAMPLES ARE **CLEARLY** DELUSIONAL IDEAS NOT EXPLORED IN QUESTIONS L1 TO L4, FOR EXAMPLE, SOMATIC OR RELIGIOUS DELUSIONS OR DELUSIONS OF GRANDIOSITY, JEALOUSY, GUILT, RUIN OR DESTITIUTION, ETC.	NO	YES	YES
	b	**IF YES OR YES BIZARRE:** do they currently consider your beliefs strange?	NO	YES	YES
L6	a	Have you ever heard things other people couldn't hear, such as voices?<br>HALLUCINATIONS ARE SCORED "BIZARRE" ONLY IF PATIENT ANSWERS **YES** TO THE FOLLOWING:	NO	YES	
		**IF YES:** Did you hear a voice commenting on your thoughts or behavior or did you hear two or more voices talking to each other?	NO		YES
	b	**IF YES OR YES BIZARRE TO L6a:** have you heard these things in the past month?<br>HALLUCINATIONS ARE SCORED "BIZARRE" ONLY IF PATIENT ANSWERS **YES** TO THE FOLLOWING:<br>Did you hear a voice commenting on your thoughts or behavior or did you hear two or more voices talking to each other?	NO	YES	YES<br>➡**L8b**

L7 a Have you ever had visions when you were awake or have you ever seen things other people couldn't see? NO YES

CLINICIAN: CHECK TO SEE IF THESE ARE CULTURALLY INAPPROPRIATE.

b **IF YES:** have you seen these things in the past month? NO YES

CLINICIAN'S JUDGMENT

L8 b IS THE PATIENT CURRENTLY EXHIBITING INCOHERENCE, DISORGANIZED SPEECH, OR MARKED LOOSENING OF ASSOCIATIONS? NO YES

L9 b IS THE PATIENT CURRENTLY EXHIBITING DISORGANIZED OR CATATONIC BEHAVIOR? NO YES

L10 b ARE NEGATIVE SYMPTOMS OF SCHIZOPHRENIA, E.G. SIGNIFICANT AFFECTIVE FLATTENING, POVERTY OF SPEECH (ALOGIA) OR AN INABILITY TO INITIATE OR PERSIST IN GOAL-DIRECTED ACTIVITIES (AVOLITION), PROMINENT DURING THE INTERVIEW? NO YES

L11 a ARE 1 OR MORE « a » QUESTIONS FROM L1a TO L7a CODED **YES OR YES BIZARRE** AND IS EITHER:

MAJOR DEPRESSIVE EPISODE, (CURRENT OR RECURRENT)
OR
MANIC OR HYPOMANIC EPISODE, (CURRENT OR PAST) CODED **YES**? NO ➡**L13** YES

IF NO TO L11 a, CIRCLE NO IN BOTH 'MOOD DISORDER WITH PSYCHOTIC FEATURES' DIAGNOSTIC BOXES AND MOVE TO L13.

b You told me earlier that you had period(s) when you felt (depressed/high/persistently irritable).

Were the beliefs and experiences you just described (SYMPTOMS CODED **YES** FROM **L1**a TO **L7**a) restricted exclusively to times when you were feeling depressed/high/irritable?

IF THE PATIENT EVER HAD A PERIOD OF AT LEAST 2 WEEKS OF HAVING THESE BELIEFS OR EXPERIENCES (PSYCHOTIC SYMPTOMS) WHEN THEY WERE NOT DEPRESSED/HIGH/IRRITABLE, CODE NO TO THIS DISORDER.

IF THE ANSWER IS NO TO THIS DISORDER, ALSO CIRCLE NO TO L12 AND MOVE TO L13

**NO**	**YES**
MOOD DISORDER WITH *PSYCHOTIC FEATURES*	
**LIFETIME**	

L12 a ARE 1 OR MORE « b » QUESTIONS FROM L1b TO L7b CODED **YES OR YES BIZARRE** AND IS EITHER:

MAJOR DEPRESSIVE EPISODE, (CURRENT)
OR
MANIC OR HYPOMANIC EPISODE, (CURRENT) CODED **YES**?

IF THE ANSWER IS YES TO THIS DISORDER (LIFETIME OR CURRENT), CIRCLE NO TO L13 AND L14 AND MOVE TO THE NEXT MODULE.

**NO**	**YES**
MOOD DISORDER WITH *PSYCHOTIC FEATURES*	
**CURRENT**	

L13 ARE 1 OR MORE « b » QUESTIONS FROM L1b TO L6b, CODED **YES BIZARRE**?

OR

ARE 2 OR MORE « b » QUESTIONS FROM L1b TO L10b, CODED **YES** (RATHER THAN **YES BIZARRE**)?

AND DID AT LEAST TWO OF THE PSYCHOTIC SYMPTOMS OCCUR DURING THE SAME 1 MONTH PERIOD?

**NO**	**YES**
PSYCHOTIC DISORDER **CURRENT**	

L14 IS **L13** CODED **YES**

OR

ARE 1 OR MORE « a » QUESTIONS FROM L1a TO L6a, CODED **YES BIZARRE**?

OR

ARE 2 OR MORE « a » QUESTIONS FROM L1a TO L7a, CODED **YES** (RATHER THAN **YES BIZARRE**)

AND DID AT LEAST TWO OF THE PSYCHOTIC SYMPTOMS OCCUR DURING THE SAME 1 MONTH PERIOD?

**NO**	**YES**
PSYCHOTIC DISORDER **LIFETIME**	

M. ANOREXIA NERVOSA

(➡ MEANS : GO TO THE DIAGNOSTIC BOX, CIRCLE NO, AND MOVE TO THE NEXT MODULE)

M1 a	How tall are you?	❑ ft ❑❑ in.	
		❑ ❑ ❑ cm.	
b.	What was your lowest weight in the past 3 months?	❑ ❑ ❑ lbs.	
		❑ ❑ ❑ kgs.	
c	IS PATIENT'S WEIGHT EQUAL TO OR BELOW THE THRESHOLD CORRESPONDING TO HIS / HER HEIGHT? (SEE TABLE BELOW)	➡ NO	YES

In the past 3 months:

M2	In spite of this low weight, have you tried not to gain weight?	➡ NO	YES
M3	Have you intensely feared gaining weight or becoming fat, even though you were underweight?	➡ NO	YES
M4 a	Have you considered yourself too big / fat or that part of your body was too big / fat?	NO	YES
b	Has your body weight or shape greatly influenced how you felt about yourself?	NO	YES
c	Have you thought that your current low body weight was normal or excessive?	NO	YES
M5	ARE 1 OR MORE ITEMS FROM **M4** CODED **YES**?	➡ NO	YES
M6	FOR WOMEN ONLY: During the last 3 months, did you miss all your menstrual periods when they were expected to occur (when you were not pregnant)?	➡ NO	YES

FOR WOMEN: ARE **M5 AND M6** CODED **YES**?

FOR MEN: IS **M5** CODED **YES**?

NO **YES**

ANOREXIA NERVOSA
CURRENT

HEIGHT / WEIGHT TABLE CORRESPONDING TO A BMI THRESHOLD OF 17.5 KG/M^2

Height/Weight

ft/in	4'9	4'10	4'11	5'0	5'1	5'2	5'3	5'4	5'5	5'6	5'7	5'8	5'9	5'10
lbs.	81	84	87	89	92	96	99	102	105	108	112	115	118	122
cm	145	147	150	152	155	158	160	163	165	168	170	173	175	178
kgs	37	38	39	41	42	43	45	46	48	49	51	52	54	55

Height/Weight

ft/in	5'11	6'0	6'1	6'2	6'3
lbs.	125	129	132	136	140
cm	180	183	185	188	191
kgs	57	59	60	62	64

The weight thresholds above are calculated using a body mass index (BMI) equal to or below 17.5 kg/m^2 for the patient's height. This is the threshold guideline below which a person is deemed underweight by the DSM-IV and the ICD-10 Diagnostic Criteria for Research for Anorexia Nervosa.

N. BULIMIA NERVOSA

(➡ MEANS : GO TO THE DIAGNOSTIC BOXES, CIRCLE NO IN ALL DIAGNOSTIC BOXES, AND MOVE TO THE NEXT MODULE)

N1	In the past three months, did you have eating binges or times when you ate a very large amount of food within a 2-hour period?	➡ NO	YES
N2	In the last 3 months, did you have eating binges as often as twice a week?	➡ NO	YES
N3	During these binges, did you feel that your eating was out of control?	➡ NO	YES
N4	Did you do anything to compensate for, or to prevent a weight gain from these binges, like vomiting, fasting, exercising or taking laxatives, enemas, diuretics (fluid pills), or other medications?	➡ NO	YES
N5	Does your body weight or shape greatly influence how you feel about yourself?	➡ NO	YES
N6	DO THE PATIENT'S SYMPTOMS MEET CRITERIA FOR ANOREXIA NERVOSA?	NO ↓ Skip to N8	YES
N7	Do these binges occur only when you are under (_____lbs./kgs.)? INTERVIEWER: WRITE IN THE ABOVE PARENTHESIS THE THRESHOLD WEIGHT FOR THIS PATIENT'S HEIGHT FROM THE HEIGHT / WEIGHT TABLE IN THE ANOREXIA NERVOSA MODULE.	NO	YES

N8 IS **N5** CODED **YES** AND IS EITHER **N6** OR **N7** CODED **NO**?

**NO**	**YES**

BULIMIA NERVOSA
CURRENT

IS **N7** CODED **YES**?

**NO**	**YES**

ANOREXIA NERVOSA
Binge Eating/Purging Type
CURRENT

O. GENERALIZED ANXIETY DISORDER

(➡ MEANS : GO TO THE DIAGNOSTIC BOX, CIRCLE NO, AND MOVE TO THE NEXT MODULE)

O1	a	Have you worried excessively or been anxious about several things over the past 6 months?	➡ NO	YES
	b	Are these worries present most days?	➡ NO	YES
		IS THE PATIENT'S ANXIETY RESTRICTED EXCLUSIVELY TO, OR BETTER EXPLAINED BY, ANY DISORDER PRIOR TO THIS POINT?	NO	➡ YES
O2		Do you find it difficult to control the worries or do they interfere with your ability to focus on what you are doing?	➡ NO	YES
O3		FOR THE FOLLOWING, CODE **NO** IF THE SYMPTOMS ARE CONFINED TO FEATURES OF ANY DISORDER EXPLORED PRIOR TO THIS POINT**.**		
		**When you were anxious over the past 6 months, did you, most of the time:**		
	a	Feel restless, keyed up or on edge?	NO	YES
	b	Feel tense?	NO	YES
	c	Feel tired, weak or exhausted easily?	NO	YES
	d	Have difficulty concentrating or find your mind going blank?	NO	YES
	e	Feel irritable?	NO	YES
	f	Have difficulty sleeping (difficulty falling asleep, waking up in the middle of the night, early morning wakening or sleeping excessively)?	NO	YES

ARE **3** OR MORE **O3** ANSWERS CODED **YES**?

**NO**	**YES**

GENERALIZED ANXIETY DISORDER
CURRENT

P. ANTISOCIAL PERSONALITY DISORDER (optional)

(➨ MEANS : GO TO THE DIAGNOSTIC BOX AND CIRCLE NO.)

P1 **Before you were 15 years old, did you:**

a	repeatedly skip school or run away from home overnight?	NO	YES
b	repeatedly lie, cheat, "con" others, or steal?	NO	YES
c	start fights or bully, threaten, or intimidate others?	NO	YES
d	deliberately destroy things or start fires?	NO	YES
e	deliberately hurt animals or people?	NO	YES
f	force someone to have sex with you?	NO	YES
	ARE **2** OR MORE **P1** ANSWERS CODED **YES**?	➨ NO	YES

DO NOT CODE **YES** TO THE BEHAVIORS BELOW IF THEY ARE EXCLUSIVELY POLITICALLY OR RELIGIOUSLY MOTIVATED**.**

P2 **Since you were 15 years old, have you:**

a	repeatedly behaved in a way that others would consider irresponsible, like failing to pay for things you owed, deliberately being impulsive or deliberately not working to support yourself?	NO	YES
b	done things that are illegal even if you didn't get caught (for example, destroying property, shoplifting, stealing, selling drugs, or committing a felony)?	NO	YES
c	been in physical fights repeatedly (including physical fights with your spouse or children)?	NO	YES
d	often lied or "conned" other people to get money or pleasure, or lied just for fun?	NO	YES
e	exposed others to danger without caring?	NO	YES
f	felt no guilt after hurting, mistreating, lying to, or stealing from others, or after damaging property?	NO	YES

ARE **3** OR MORE **P2** QUESTIONS CODED **YES**?

NO **YES**

ANTISOCIAL PERSONALITY DISORDER
LIFETIME

THIS CONCLUDES THE INTERVIEW

REFERENCES

Sheehan DV, Lecrubier Y, Harnett-Sheehan K, Janavs J, Weiller E, Bonara LI, Keskiner A, Schinka J, Knapp E, Sheehan MF, Dunbar GC. Reliability and Validity of the MINI International Neuropsychiatric Interview (M.I.N.I.): According to the SCID-P. European Psychiatry. 1997; 12:232-241.

Lecrubier Y, Sheehan D, Weiller E, Amorim P, Bonora I, Sheehan K, Janavs J, Dunbar G. The MINI International Neuropsychiatric Interview (M.I.N.I.) A Short Diagnostic Structured Interview: Reliability and Validity According to the CIDI. European Psychiatry. 1997; 12: 224-231.

Sheehan DV, Lecrubier Y, Harnett-Sheehan K, Amorim P, Janavs J, Weiller E, Hergueta T, Baker R, Dunbar G: The Mini International Neuropsychiatric Interview (M.I.N.I.): The Development and Validation of a Structured Diagnostic Psychiatric Interview. J. Clin Psychiatry, 1998;59(suppl 20):22-33.

Amorim P, Lecrubier Y, Weiller E, Hergueta T, Sheehan D: DSM-III-R Psychotic Disorders: procedural validity of the Mini International Neuropsychiatric Interview (M.I.N.I.). Concordance and causes for discordance with the CIDI. European Psychiatry. 1998; 13:26-34.

**Translations**	**M.I.N.I. 4.4 or earlier versions**	**M.I.N.I. 4.6/5.0, M.I.N.I. Plus 4.6/5.0 and M.I.N.I. Screen 5.0:**
Afrikaans	R. Emsley	W. Maartens
Arabic		O. Osman, E. Al-Radi
Bengali		H. Banerjee, A. Banerjee
Braille (English)		
Brazilian Portuguese	P. Amorim	P. Amorim
Bulgarian	L.G.. Hranov	
Chinese		L. Carroll, Y-J. Lee, Y-S. Chen, C-C. Chen, C-Y. Liu, C-K. Wu, H-S. Tang, K-D. Juang, Yan-Ping Zheng.
Czech		P. Zvlosky
Danish	P. Bech	P. Bech, T. Schütze
Dutch/Flemish	E. Griez, K. Shruers, T. Overbeek, K. Demyttenaere	I. Van Vliet, H. Leroy, H. van Megen
English	D. Sheehan, J. Janavs, R. Baker, K. Harnett-Sheehan, E. Knapp, M. Sheehan	D. Sheehan, R. Baker, J. Janavs, K. Harnett-Sheehan, M. Sheehan
Estonian		J. Shlik, A. Aluoja, E. Khil
Farsi/Persian		K. Khooshabi, A. Zomorodi
Finnish	M. Heikkinen, M. Lijeström, O. Tuominen	M. Heikkinen, M. Lijeström, O. Tuominen
French	Y. Lecrubier, E. Weiller, I. Bonora, P. Amorim, J.P. Lepine	Y. Lecrubier, E. Weiller, P. Amorim, T. Hergueta
German	I. v. Denffer, M. Ackenheil, R. Dietz-Bauer	G. Stotz, R. Dietz-Bauer, M. Ackenheil
Greek	S. Beratis	T. Calligas, S. Beratis
Gujarati		M. Patel, B. Patel, Organon
Hebrew	J. Zohar, Y. Sasson	R. Barda, I. Levinson, A. Aviv
Hindi		C. Mittal, K. Batra, S. Gambhir, Organon
Hungarian	I. Bitter, J. Balazs	I. Bitter, J. Balazs
Icelandic		J.G. Stefansson
Italian	I. Bonora, L. Conti, M. Piccinelli, M. Tansella, G. Cassano, Y. Lecrubier, P. Donda, E. Weiller	L. Conti, A. Rossi, P. Donda
Japanese		T. Otsubo, H. Watanabe, H. Miyaoka, K. Kamijima, J.Shinoda, K.Tanaka, Y. Okajima
Kannada		Organon
Korean		K.S. Oh and Korean Academy of Anxiety Disorders
Latvian	V. Janavs, J. Janavs, I. Nagobads	V. Janavs, J. Janavs
Lithuanian		A. Bacevicius
Malayalam		Organon
Marathi		Organon
Norwegian	G. Pedersen, S. Blomhoff	K.A. Leiknes , U. Malt, E. Malt, S. Leganger
Polish	M. Masiak, E. Jasiak	M. Masiak, E. Jasiak
Portuguese	P. Amorim	P. Amorim, T. Guterres
Punjabi		A. Gahunia, S. Gambhir
Romanian		O. Driga
Russian		A. Bystritsky, E. Selivra, M. Bystritsky, L. Shumyak, M. Klisinska.
Serbian	I. Timotijevic	I. Timotijevic
Setswana		K. Ketlogetswe
Slovenian		M. Kocmur, M. Kocmur
Spanish	L. Ferrando, J. Bobes-Garcia, J. Gilbert-Rahola, Y. Lecrubier	L. Ferrando, L. Franco-Alfonso, M. Soto, J. Bobes-Garcia, O. Soto, L. Franco, G. Heinze, C. Santana, R. Hidalgo
Swedish	M. Waern, S. Andersch, M. Humble	C. Allgulander, H. Agren M. Waern, A. Brimse, M. Humble.
Tamil		Organon
Telugu		Organon

Thai		P. Kittirattanapaiboon, S. Mahatnirunkul, P. Udomrat, P. Silpakit,, M. Khamwongpin, S. Srikosai.
Turkish	T. Örnek, A. Keskiner, I. Vahip	T. Örnek, A. Keskiner, A.Engeler
Urdu		S. Gambhir

A validation study of this instrument was made possible, in part, by grants from SmithKline Beecham and the European Commission.
The authors are grateful to Dr. Pauline Powers for her advice on the modules on Anorexia Nervosa and Bulimia.
