# Supplementary material for: An exploration of the conditions for deploying self-management strategies: a qualitative study of experiential knowledge in depression
Source: BMC Psychiatry. 2020 May 11;20:210. doi: 10.1186/s12888-020-02559-3 (PMC7212680; doi:10.1186/s12888-020-02559-3)
Supplement: Supplementary file 3 — Additional file 3. Interview guide. [file 12888_2020_2559_MOESM3_ESM.docx]

| **Interview guide research article:** "An exploration of the conditions for deploying self-management strategies: a qualitative study of experiential knowledge in depression”.  By: Dorien Smit (M.Sc.)*, Janneke Peelen (Ph.D.), Janna Vrijsen (Ph.D.), and Jan Spijker (MD, Ph.D.)  *Mental Health Care Pro Persona, Department Research, Program for Mood Disorders, Centre of Expertise Depression.*  *Behavioral Science Institute, NijCa²re, Radboud University, Nijmegen, The Netherlands.*  ** Correspondence:* [*d.smit@propersona.nl*](mailto:d.smit@propersona.nl) | |
| --- | --- |
| **Introduction** | |
| **Course of depression** | *Can you tell me something about the course of your depression?*   - Start - Triggers/process - Current state of mind - Current therapy |
| **Questions per topic** | |
| **Coping with depression** |  |
| **The self** | - *What was helpful in coping with depression?* - Personal characteristics - Activities, daily structure - Asking for help - Acknowledging the problem - Triggers/signals - *In your experience, what is the role of self-reflection in coping with depression?* - *Can you describe your attitude towards depression?* - Did your attitude change over the course of time, with regard to acceptance? - *Do you experience different phases in coping with depression?* - *What did you need to deploy self-management strategies?* |
| **Gaining insight** | - *Did you change your way of managing depression over the course of the illness?* - *What contributed to the development of personal fitting coping strategies?* - *What was helpful to gain insight in your (behavior and/or thought) patterns?* - The role of mental health care in gaining insight - Changes in coping with depression after these new understanding - *According to your experience, in what manner is experiential knowledge of coping with depression developing?* |
| **Environment** | - *What did you learn from others about coping with depression?* - *What is the role of your social network in coping with depression?* - Family/friends - Work - Peer support - Societal context - *In what manner did your social network help you when coping with depression?* - What would have helped? - What was not helpful? |
| **Religion/spirituality** | - *What is the role of religion or spirituality in coping with depression for you?* - Existential questions - Giving meaning |
| **Professional help** | - *In your experience, what is the role of professional help in coping with depression?* - Mental health care, therapy - Medication - Counselling - Alternative therapies - Follow-up care |
| **Role of depression in life** | - *What are positive and negative aspects of suffering from depression, in your experience?* |
| **Experiential expertise** | - *What is your opinion about experiential expertise of depression?* - Personal experience of being counselled by an experiential expert - Role of the patient organisation: Dutch Depression Association - Risks versus added value of experiential expertise in mental health care for depression |
| **Completing the interview** | - *Do you have anything to add to this conversation?* - *Did we miss something, topics that we did not discuss, but are important to you in coping with depression?* |
